# Digitizing microscope slide-based natural history collections: A protocol using slide scanner technology

**DOI:** 10.1371/journal.pone.0346139

**Published:** 2026-04-24

**Authors:** Ingrid C. Romero, Scott L. Wing, Carlos A. Jaramillo, Holly Little, Surangi W. Punyasena, Alexander E. White

**Affiliations:** 1 Department of Paleobiology, Smithsonian National Museum of Natural History, Washington, District of Columbia, United States of America; 2 Smithsonian Tropical Research Institute, PanamaPanama; 3 Department of Plant Biology, University of Illinois Urbana-Champaign, Urbana-Champaign, Illinois, United States of America; 4 Smithsonian Office of Digital and Innovation, Washington, District of Columbia, United States of America; Sun Yat-Sen University School of Geography and Planning, CHINA

## Abstract

Natural history collections contain millions of microscope slides documenting global microscopic biodiversity, yet these materials remain largely undigitized and are vulnerable to deterioration and loss. Recent advances in slide scanner technology, originally developed for medical pathology, offer new opportunities for comprehensive digitization of slide-based collections. Here we present an optimized protocol for digitizing diverse microscope slide specimens, using the Hamamatsu NanoZoomer S20 slide scanner, developed while imaging slides at the Smithsonian National Museum of Natural History. We provide specimen-specific recommendations for scanning parameters, including scan area, focal points, Z-stack configuration, and file management workflows. Scanning times range from 41 seconds for small invertebrates to 18 minutes for palynological samples, with final compressed file sizes of 0.15-28 GB. High-resolution images (0.23 μm/pixel) captured diagnostic morphological features across all specimen types, including pollen, diatoms, radiolarians, plant and fungi tissues, and invertebrates. Using this method, we estimated that just the NMNH’s paleo-palynology slide collection contains approximately 4.3 billion individual specimens, 30 times more than the current estimated size of the entire NMNH collection. Slide scanning enables 3D data capture, facilitates remote collaboration, improves reproducibility of taxonomic identifications, and creates permanent digital records that mitigate risks of physical deterioration. This protocol provides practical guidance for institutions looking to digitize slide-based collections to preserve and unlock their full research potential.

## Introduction

Natural history collections (NHC) contain extensive biodiversity data that provide unique historical records across broad taxonomic, geographic, and temporal scopes [1–3]. They also encapsulate decades, if not centuries, of dedicated effort by countless researchers, collectors, and naturalists [4,5]. Despite their value, these collections are susceptible to deterioration over time due to environmental factors, including fluctuations in temperature, humidity, and exposure to light [6,7]. This degradation affects not only large specimens but also microscope slide-based specimens such as palynological samples and siliceous microfossils [7]. Collections of this type, such as the USGS-Denver Pollen Collection (DPC) housed in the Department of Paleobiology at the Smithsonian National Museum of Natural History (NMNH), often exhibit visible signs of deterioration, including yellowing, cracking, crystallization, and detachment of cover slips [7,8], all of which can render specimens unidentifiable or cause permanent loss.

In the last two decades, digitization efforts have intensified, with more than 140 million specimens now digitized and accessible through the Global Biodiversity Information Facility (GBIF) [9], preserving digital representations of collection material and allowing researchers to access metadata and materials without handling physical specimens. Digitization also opens new avenues for data-driven research, such as the use of convolutional neural networks to quantify morphological characters, allowing for ecological and evolutionary inferences [10–12]. Most digitization efforts have been focused on macro-specimens, such as vertebrates [13,14], invertebrates [15,16], and herbarium specimens [17,18]. Billions of smaller specimens mounted on microscope slides remain undigitized, with individual slides often containing thousands of specimens [19].

Imaging microscope slides creates permanent digital records of these specimens and reduces the risk of physical damage from accidental drops, mishandling, or transport. Significant advancements in microscope slide scanners offer new opportunities for digitizing and preserving NHC stored on microscope slides. These scanners enable the imaging of smaller specimens, ranging from micrometers (µm) to millimeters (mm), a task previously limited by size constraints [20,21]. While these scanners have traditionally been used in medicine, their application in biological and paleontological research has expanded, enabling high-resolution and rapid imaging of microscope slides [19,22–25].

Here, we present a comprehensive protocol for imaging NHC using slide scanner technology. We describe optimized scanning parameters for diverse specimen types, provide workflows for file management and analysis, and demonstrate how this technology enhances collections accessibility and enables new research applications. We developed this protocol using the Hamamatsu NanoZoomer S20 slide scanner (NZ) at the Smithsonian NMNH, but the principles apply broadly to other slide scanning systems. We also review the advantages of slide scanning for digitizing and preserving slide collections.

## Materials and methods

### Slide scanner technology

Microscope slide scanners comprise a precision motorized stage for slide movement, a high-quality objective lens, a light source, a digital camera for image acquisition, and specialized software for image visualization and analysis [26]. The slide scanner stage moves the slide while a camera captures tiled images as the scan progresses. This process aims to generate a seamless, high-resolution image of the entire slide [26]. These scanners enable imaging of specimens ranging in size from micrometers (µm) to millimeters (mm) within seconds to a few minutes. Most slide scanners, like the NZ, are designed for standard pathology slides (75 x 25 x 1 mm), the most common slides used to mount specimens in biological collections and some paleontological collections. Some scanners also have features that allow the scanning of samples mounted in slides with different dimensions (e.g., Zeiss Axioscan and Olympus VS200 for slides 1x3”, 2x3”, 4x3”), commonly used in geological fields [27].

**Fig 1:**
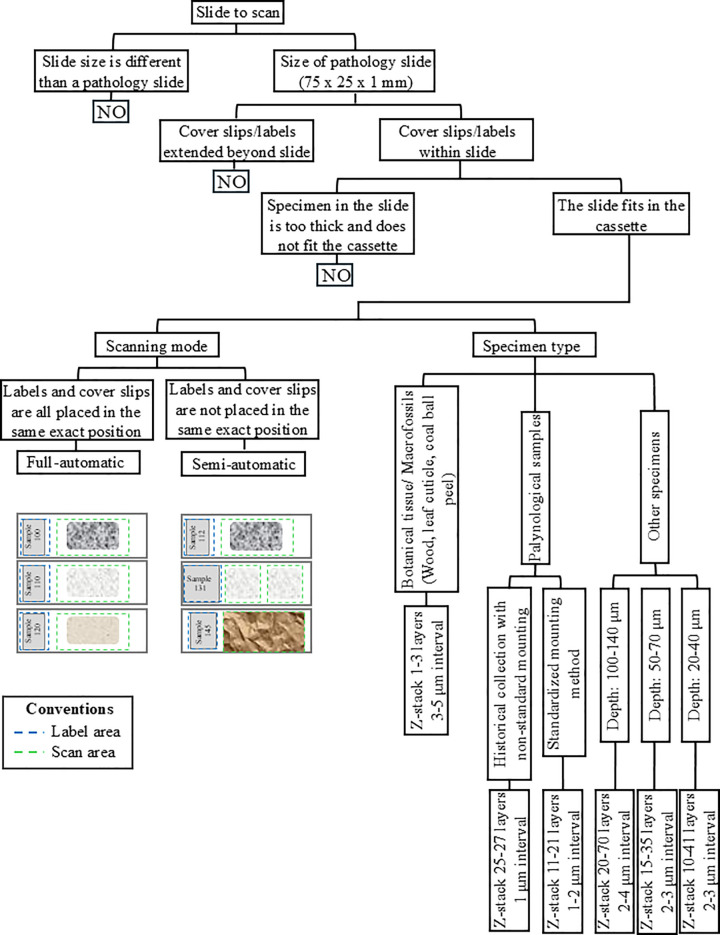
Flowchart outlining the steps required to determine which microscope slides are suitable for placement on the NZ system and to successfully complete image capture, based on the characteristics of both the slide and the specimen. The label and scan areas delimited by dashed lines illustrate the types of slides that are best scanned in each mode.

A major proportion of slides in historical collections at the NMNH, such as those in the DPC, were prepared on standard microscope slides following various methods for pollen preparation. To support the digitization of the DPC (70,000 slides, roughly 25,500 from cores and outcrops in the continental U.S., 40,000 from Alaska, and 4,500 from other parts of the world), the NMNH recently acquired a NZ, characterized by high-speed, high-resolution imaging and a compressed file format that produces manageable file sizes [28,29]. Here, we share our protocol for optimizing the scanning of slide-mounted specimens in historical collections, developed after imaging over a thousand slides [30]. To illustrate variations in scanning settings and the capabilities of these digital scanners, we selected 14 representative specimens (S1 Table, Fig 1). The protocol described in this peer-review article is published on protocols.oi, https://dx.doi.org/10.17504/protocols.io.bp2l6ex61gqe/v1 [30] and is included for printing as supporting information File 1 with this article. Although this study focuses on the NZ scanner, the protocol is broadly applicable to other slide scanners of comparable quality and cost, including the Zeiss Axioscan, 3DHistech Pannoramic, Leica Aperio GT 180, Nikon BioPipeline, Olympus SLIDEVIEW VS200, and Morphle MorphoLens 240. Additionally, no permits were required for the described study, which complied with all relevant regulations.

### Preparation for scanning

Before scanning, slides should be reviewed to ensure scanner compatibility and prevent slide breakage. The cover slip and label must be affixed to the same side of the slide, with nothing attached to the back of the slide or extending beyond the edges. The label must lie completely flat. Additionally, the NZ’s autofocus works best when the cover slip is coplanar with the slide surface. In NHC, slides should have assigned collection identifiers, e.g., catalog numbers, visible on the slide. These numbers ensure that slides can be properly tracked and associated with the metadata, images, and records for that object or specimen. Catalog numbers also allow for easy search and discovery of specimens and images.

Creating a digital archive of microscope slides requires standard naming practices. Naming protocols should be established before any large-scale digitization effort. Properly naming digital files is essential when imaging NHC, as the NZ saves files automatically in.ndpi (NanoZoomer Digital Pathology Image) format after scanning. If a file naming format is not established beforehand, NDPI files are saved using the date and time when the file was created. The NZ has linear image sensors that can read 1D and 2D barcodes, which can be used to automate file naming during the scanning process, ensuring the specimens’ unique names and collection identifiers are captured, and reducing errors in transcribing specimen data.

### Scanning settings

**Fig 2:**
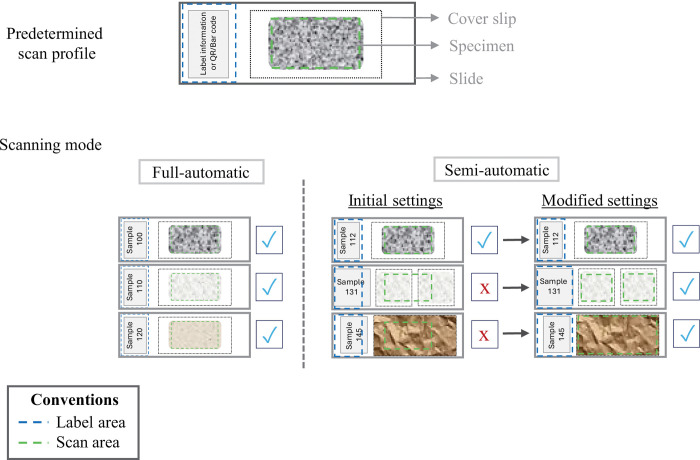
Scanning modes based on a predefined profile, in which the label area and scan area are predetermined.

The NZ scanner offers two batch modes: fully automatic and semi-automatic. In fully automatic mode, the user selects the preferred profile for the set of slides to scan. When the scanning starts, all selected slides are imaged continuously and without stopping (Figs 1, 2). This mode can also be set for the scanner to autodetect the specimen. The fully automatic mode is suitable for slide collections in which labels and cover slips are placed in the exact same position across all slides, but NHCs are typically too heterogeneous (Figs 1, 2). The semi-automatic mode is more appropriate for NHC because it allows manual review and adjustment of the label and scan area for each slide, when necessary, and before the scanning begins (Figs 1, 2). In this mode, reviewing the settings of each slide, a 20-slide batch can take approximately 5–20 minutes. However, during this time, the scanning is already in process because as soon as the first slide is set, the scanning starts. Thus, the following slides will scan sequentially. For scanning and imaging of the slides, we used Hamamatsu’s NZAcquire software version 3.1.20 [30]. To visualize the final images, we used the Hamamatsu NDP.view2 software [31] (S1, S2 Videos).

### Scanning parameters: optimization by specimen type

When configuring the imaging profile in NZ it is important to consider several parameters, including scan area, split tissue setting, number and positions of focal points, and the number and spacing of Z-stack layers. The settings of the parameters depend on the specimen type and are explained in more detail below. The NZ system uses a 20X objective with a precision optical coupler that allows scanning in two modes, 20X mode with a 0.46 μm/pixel resolution and 40X mode with 0.23 μm/pixel resolution (S1 File). This feature has the advantage that the time of scanning in both modes is similar (i.e., scanning speed of a 15 mm x 15 mm area will take approximately 30s in both 20X and 40X modes). Specimens such as insects, wood tissue, and coal ball peels can be imaged using either mode, 20X or 40X, while for microfossils, such as pollen, diatoms, and radiolarians, 40X is preferred. All specimens figured for this paper were scanned using the 40X mode because it provides better resolution with little additional time in scanning.

### Scan area

**Fig 3:**
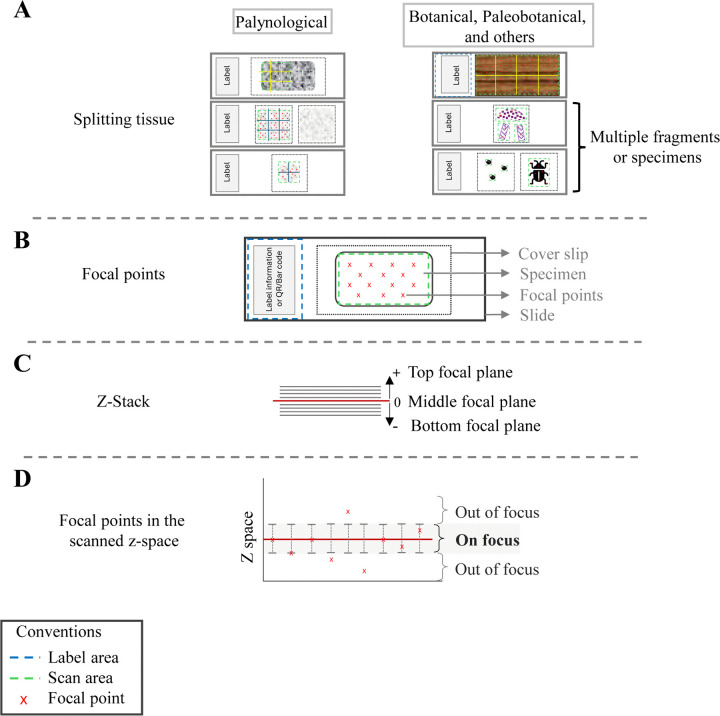
Scanning settings. **A)** Split tissue configuration based on the type of sample or specimen. **B)** Placement of focal points within a scan area. **C)** Representation of the Z-stack showing the middle plane (red) and an equal number above and below the middle plane (black). **D)** Illustration of the focal points in Z-space demonstrating how some areas of the slide may appear out of focus in the final digital image.

The size of the selected scan area depends on the type of sample or specimen being imaged (Fig 3A). For samples with microfossil specimens (pollen, spores, dinoflagellates, radiolarians, including diatoms and phytoliths), which have dozens to thousands of individual specimens per slide, we recommend selecting one or two scan areas of approximately 20 x 20 mm, depending on the cover slip size. This is generally sufficient to image most of the specimens, while keeping file sizes manageable for analysis (< 30Gb; Fig 3A), and facilitates storage, management, and sharing of files among collaborators. This is particularly important when sharing files with colleagues from different institutions, where transfer speeds and storage conditions may vary.

For larger specimens (>0.5 µm) such as cuticles, wood samples, fungal tissue, coal ball peels, and small invertebrates (e.g., ostracods and insects), the size of the scan area varies, based on the size and/or number of specimens present on the slide (Fig 3A). We recommend avoiding scanning the edges of the cover slip as this can interfere with the scanner’s ability to detect and focus on the specimen.

### Focus points

This feature works as quality control for appropriate specimen detection and to prevent scan failures caused by dirt or debris on the slides [32]. The focal points are initially set automatically when defining the profile settings. To set the focal point option, the NZAcquire software has three options available per scan area: 1, 5, or 9 focus points (S1 File). The number of points recommended depends on the scan area size (Fig 3A). However, even when a specific number is selected, the NZ may automatically adjust the number of focal points based on material detection, and in those cases, these points can be manually modified or moved. Thus, before imaging, the NZ scans the entire slide and generates a focus score for each checkpoint (focus point) and displays them on the monitor for quality review (Fig 3B, D). The score is represented by color-coding each focus point: light green (in focus), dark green (possibly in focus), and red (out of focus). If most of the focus points are red or dark green, the scanner may not be able to proceed with image capture. In this case, focus points can be set manually to improve focus.

For microfossil slides with 20 mm x 20 mm scan areas, we recommend five focal points per subdivided scan area (see *Split tissue section*, Fig 3A); larger scan areas require nine points. Autofocus success depends critically on the method of slide preparation. Automatically placed focal points typically produced sharp images if the microfossils were applied to the coverslip, which was then inverted and affixed to the slide with mounting medium of uniform thickness (e.g., Amazon Drilling Project samples; Fig 3). In contrast, manual focal point adjustment was typically required for historical collections such as the DPC, where material was mixed with mounting medium and then applied to the slides. The scanner’s automatic focal points often failed to detect material or lost focus because specimens were distributed unevenly across focal planes, and/or the mounting medium had deteriorated (dried and shattered), and the coverslip had deformed. Manual focus is also recommended for slides in which the mounting medium has shattered but the microfossils are preserved in good condition and can still provide important information (Fig 3).

We do not recommend scanning barren microfossil slides, slides with very few specimens, or slides in which the mounting medium is shattered and the microfossils are destroyed, because the information preserved is not likely to justify the time, effort, and file storage space. We do recommend recording the reason a given slide was not scanned in case future improvements in imaging technology change the cost-benefit calculation. To rapidly assess whether a slide is suitable for digitization, it is important to ensure that the mounting medium is not deteriorated or severely degraded. When viewed under transmitted light, the slide should appear slightly cloudy due to particulate organic matter. A brief inspection under an optical microscope should also confirm the presence of multiple specimens in at least five fields of view.

For larger specimens (>0.5 µm), the optimal number of focal points will vary based on the size of the specimen. For specimens that occupy the entire cover slip, using the “split tissue” function (see next section) is critical, as well as assigning five or nine focal points per subregion (Fig 3A). For smaller fragments, such as cuticles, five points are generally sufficient, but the number may be adjusted on the scan area (Fig 3A).

### Split tissue

The NDP-acquisition software can detect and focus on multiple sub-scan areas (tissue pieces in the context of scanning medical specimens) within a single scan area. This setting is also available in other slide-scanners under similar names and parameter settings. “Split tissue” is particularly helpful when dealing with unevenly mounted material, as it improves the likelihood of obtaining a uniformly focused image. This setting defines the maximum size (in mm) of the tissue piece that will be focused on using a single focal plane (Fig 3A). When the scan area is larger than the size specified in the split tissue setting (usually 5 mm), the software automatically divides it into smaller subregions and applies separate focal planes for each sub-scan area (Fig 3A, S1 File). For microfossils as well as specimens with scan areas greater than 10 x 10 mm^2^, we recommend using the automated split function to divide the scanned area into several equal-sized subareas. This increases the likelihood that most of the material will be in focus (Fig 3A). For slides with multiple large fragments or specimens (>0.5 µm), we recommend manually drawing scan areas around each specimen, rather than relying solely on automatic subregion detection. This allows the size of the scan area to vary based on the size of each specimen/fragment, improving focus (Fig 3A).

### Z-stack

This feature captures 3D structures by imaging across multiple focal planes [24,33]. The NZ can capture Z-stacks up to 300 µm deep. Within this limit, the actual scanned depth depends on both the number of layers (ranging from 1 to 301) and the spacing between the layers, which can vary from 1 to 150 µm (Fig 3C, 3D). The Z-stack settings for the specimens presented here showed that the number of layers and spacing between them varied based on the specimen size and the detail of morphological information that can be captured in the Z-space (*see more details below*, Table 1). It is important to highlight that the number of layers selected is always an odd number because the middle layer is the central focal plane, which is set using the focus points, then the layers above (+) and below (-) are numbered serially. For example, if 15 layers are scanned, the microscope will image the middle layer plus seven above (layers +1 to +7) and seven below (layers −1 to −7; Fig 3C, 3D).

**Table 1:** Scanning settings, timing of scanning, size of image, and final digital file of 13 selected specimens. All specimens were imaged on a 40x magnification objective. Scanning time refers to the time the NZ took to focus, scan the slide, and write the digital file. This time varies depending on the size of the selected scanned area, the number of z-stack layers, and the number of focal points.

Specimen type	Modern/Fossil	Area Scanned (mm)	Z-stack	Interval between Layers (um)	Focus range (um)	Scanning Time	Original image size (GB)	Final file Size (GB)
1. Pollen	Fossil	20.2 x 20.6	25	1	−12–12	18 m 37 s	554	27.9
2. Diatom	Modern	19.3 x 20 `	15	1	−7–7	10 m 24 s	309	19
3. Radiolarian	Fossil	20.2 x 20.6	21	1	−10–10	15 m 59s	465	19.2
4.Fungi	Fossil	18.5 x 15.3	21	1.5	−15–15	10 m 56 s	316	24.4
5. Cuticle	Fossil	10.5 x 8.96	11	1	−5–5	2 m 20 s	55.4	3.26
6. Coal ball peel	Fossils	13.2 x 13.2	5	2	−4–4	1 m 52 s	46.3	3.56
7. Wood tissue	Modern	12.3 x 16.9	3	15	−15–15	1 m 22 s	33.2	1.95
8. Ostracod	Fossil	0.879 x 1.05	71	2	−70–70	41 s	3.5	0.149
9. Ostracod	Fossil	1.76 x 1.58	71	2	−70–70	1 m 10 s	10.5	0.424
10. Flea	Modern	5.27 x 5.27	75	4	−148–148	6 m 13 s	111	3.96
11. Flea	Modern	7.03 x 4.22	75	4	−148–148	5 m 50 s	118	4.27
12. Whitefly	Modern	7.91 x 3.16	41	1	−20–20	2 m 39 s	54.7	2.09
13. Coleoptera	Modern	3.52 x 2.11	81	3	−120–120	2 m 18 s	32	1.24
14. Coleoptera	Modern	4.39 x 3.69	81	3	−120–120	4 m 22 s	70	2.93

For microfossils, the optimal Z-stack configuration depends on the depth of individual microfossils and how they are distributed along the Z-axis. For some slides in the DPC, the sample concentrate was first mixed with the mounting medium, then placed on the slide, causing the specimens to be dispersed along the Z-axis. For these slides, a Z-stack of 25–31 layers at 1 µm intervals has proven effective in capturing not only the three-dimensional structure of individual pollen grains but also most of the grains on the slide (Table 1). For other slides in which all specimens are in a similar Z-plane because the concentrate was dried onto the underside of the cover slip before mounting, a Z-stack comprising 15–21 layers at 1 µm intervals may be sufficient to capture the full depth of the material along the Z-axis. Previous studies have used different settings to scan microscope slides. In Punyasena et al. [23], for example, they scanned 150 modern pollen slides from Barro Colorado Island (BCI), Panama, using a Z-stack of 41 layers at 1 µm. This was one of the first studies using slide scanners for tropical palynological samples, characterized by the high abundance and diversity of pollen grains. Feng et al. [25] scanned Z-stacks of seven to nine layers at 3–4 µm intervals in a project involving 19 pollen slides from the Holocene of Ecuador. Here, the authors were interested in using AI models for pollen identification, in which fewer focal planes are sufficient for training the models. For digitization of microfossils from NHC that will serve as digital surrogates of physical specimens, we recommend spacing between layers of 1µ, and between 21–30 layers, depending on the type of microfossils and the distribution of the fossils in the Z-space.

For larger specimens (>0.5 µm)**,** the number of z-stack layers varies with the size of the specimen (Table 1). Most samples of leaf cuticles, wood and fungi sections, and coal ball peels were scanned with three to five planes spaced three to five µm apart on the Z-axis; this produced focused images in spite of undulations in the specimens. We varied the number of focal plane layers from 31 to 81 for various fleas, beetles, and ostracods according to the depth of the specimens. The intervals between layers also varied among specimens, with 3–4 µm intervals capturing enough morphological detail for different body parts of insects, while for ostracods and smaller insects, such as white flies, 2 µm intervals between layers captured morphological details in better focus (Table 1).

### Timing of scanning

The scanning times reported in Table 1 include focusing, scanning, and file writing. The duration of each of these processes is recorded in the final NDPI file. Focusing time is the period during which the NZ reviews each focal point to locate the material to be scanned and to define the middle focal plane (Fig 3C). This time depends on the total number of focal points per slide (Fig 3B). Scanning time corresponds to the time the NZ spends scanning a single slide and depends on the selected scan area and the number of focal planes (Figs 2, 3). Writing time refers to the period during which the software generates the final NDPI file.

Manual operation of the NZ, which includes loading and unloading slides, setting the scanning profile, and replacing slides, requires approximately 5–20 minutes. However, as mentioned in the *Scanning settings* section, once the settings for the first slide are finalized, scanning can begin while the parameters of the remaining slides are reviewed and adjusted. Therefore, this manual effort does not delay the scanning process, as it can be performed in parallel with scanning.

### Management of NDPI files

#### Image format for long-term data preservation.

The NDPI files (.ndpi extension) generated by the NZ, use a TIFF-like structure but differ in that each file includes a stack of multiple images (Z-stack layers). The NDPI format also supports image files larger than 4 GB [34]. Additionally, NDPI files are compressed in a lossy format by the scanner software, creating final files that contain all the metadata but are less than 10% of the original image size (Table 1). Thus, the final size of the files from the specimens imaged here ranged from a few megabytes (MB) to nearly 30 gigabytes (GB; Table 1). These file sizes facilitate the management, analysis, and sharing of the NDPI files. Although NDPI files contain all the original metadata and three-dimensional properties crucial for future digital microscopy analyses, these files are proprietary to Hamamatsu, and their long-term accessibility depends on continued vendor support. Similar limitations apply to other proprietary file formats (PFFs) used in whole-slide imaging (WSI), such as SVS and CZI. These formats pose challenges related to accessibility, interoperability among PFFs, reproducibility, and data analysis, making them not optimal for long-term preservation of NHC digital archives [35–37].

In response to the rapid increase of PFFs files, the Open Microscopy Environment (OME) consortium has developed open-source tools, such as the Bio-Formats library, to standardize the translation, conversion, and interoperability of PFFs [35,38–40]. In parallel, several open formats have been developed to address the challenges posed by WSI data, including Open Microscopy Environment-TIFF (OME-TIFF) [35,41], Hierarchical data format (HDF5) [39,42], and Zarr [39,43].

Considering these factors, we recommend saving files in their original proprietary format as well as in an open-access New Generation File Format (NGFF), such as Zarr or HDF5. These standardized, open formats are designed to support scalable access to large, high-resolution, multi-dimensional data and associated metadata [36,41]. This recommendation reflects still-evolving best practices for long-term preservation of WSI. Although OME-TIFF was among the first formats developed to address WSI challenges, it presents limitations when scaling pyramidal resolution images to multi-dimensional datasets and often results in file sizes larger than the original WSI file [39,44]. NGFF formats are therefore a more suitable option for long-term storage of WSI files within NHC digital archives, as they better preserve the full complexity of WSI data.

In addition to these analysis-ready formats, we recommend generating a master Tagged Image File Format (TIFF) derivative for archiving within institutional systems of record (e.g., Digital Asset Management Systems) and for integration with primary specimen data maintained by NHC. Although TIFF files do not preserve the full multi-resolution structure of a WSI or all the metadata from the original file [44], they are commonly recommended for long-term preservation and backup of digital NHC archives because they are universally readable, relatively stable, and aligned with established data mobilization and preservation practices [43]. Together, the use of NGFF files to maintain data complexity and TIFF derivatives to support institutional workflows, accompanied by clear descriptive metadata documenting their relationships, provides a balanced approach to long-term preservation, access, and reuse of WSI data.

### Access, analysis, and sharing of WSI

Several free and open-access software tools can be used for managing and analyzing the WSI files. NDP.view2^®^ is specially designed for digital slide observation of NDPI images and analysis [31]. ImageJ Fiji has different tools that can convert PFFs to standard TIFF format; for NDPI files, ImageJ Fiji has the NDPITools ImageJ plugin [45]. OpenSlide is a C library that provides a simple interface for reading whole-slide images [46] and it can be used through different interfaces or platforms, such as Python with OpenSlide-Python, through OMERO (Open Microscopy Environment), and QuPath (Open Software for Bioimage Analysis).

Sharing and viewing large WSI files among collaborators, institutions, and collections users is a challenge even with fast networks and large storage devices. We have used two primary tools for sharing files, Globus and OMERO. Globus is a file transfer service that provides reliable data transfer between workstations, high-performance computer servers, data repositories, user devices, and endpoints [47]. It excels in high-speed transfers and allows for data stream interruptions [47]. Unlike cloud-storage services such as Dropbox, Globus allows centralized control over data and minimizes concerns about file loss risks during transfers, making it especially reliable for large-scale data sharing. OMERO is a free, open-source, image data management interface for viewing files on a remote server [20,48]. OMERO allows multiple scientists to simultaneously visualize and analyze the image files, facilitating not only accessing and processing, but also sharing of large image data and metadata [20,48]. OMERO also allows users to analyze the images without having to download the large, original files because these are maintained by the institution housing the files [20,24,48].

Digitizing slide collections requires fast network speeds and large storage devices for working with large images. Some institutions may find that placing a slide-scanner, such as the NZ, on their local network is difficult because of local security requirements. The computer that controls NZ scanning is imaged by Hamamatsu and cannot be reimaged by the end user. Security rules may not permit workstations that have not been imaged locally to be added to the museum or university network. NDPI scan files are so large that it can take many hours to copy them to an external drive, then upload them to networked servers. To overcome this, we connected the NZ computer to an internally networked server behind the institutional firewall, then mounted it to the internal Globus server. This setup enabled file transfer from the NZ computer to network-connected resources without the need for copying to an intermediate external drive, facilitating subsequent sharing with internal and external collaborators.

## Results

**Fig 4:**
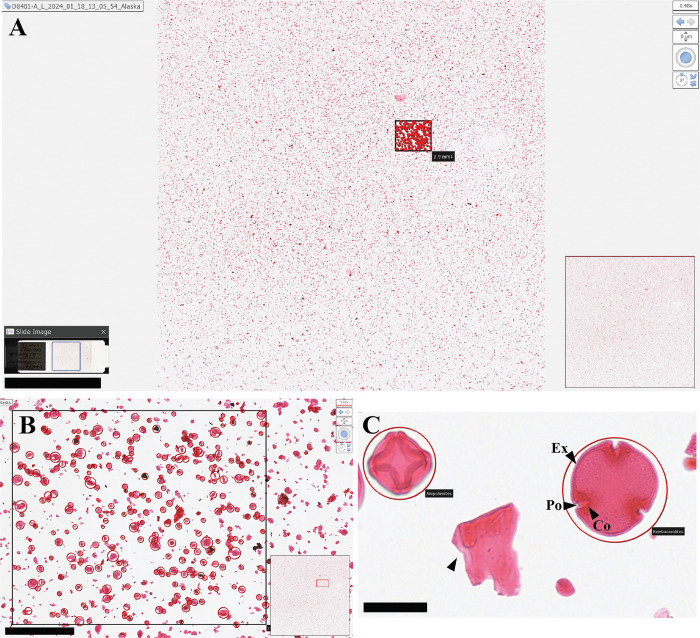
Digital images of a fossil pollen slide from the Miocene (11−5 Ma) of Alaska, part of the DPC (sample D8401) housed at NMNH Paleobiology. **A)** Complete image of the slide (lower left) with scan area in a blue box. Main image shows a square scanned area with a small rectangle indicating the area enlarged in **B. B)** Magnified area in rectangle with identified and annotated grains indicated by red circles. **C)** Magnified view from within area B showing two pollen grains, *Alnus* (left) and Bombacoideae (right). Ex – Exine, Po – Pore, Co – Colpus. Bar scales: (A) 5 mm, (B) 500 µm, (C) 25 µm.

**Fig 5:**
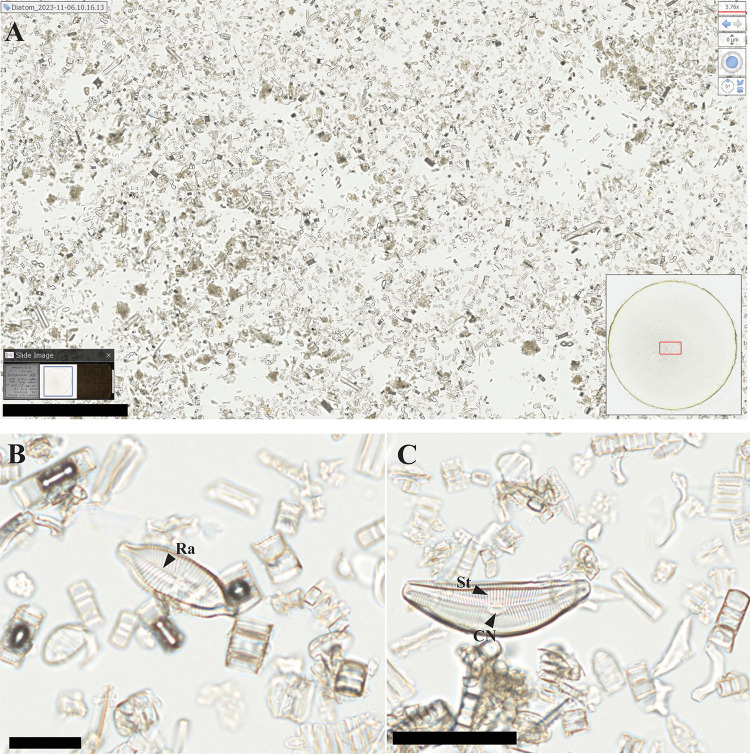
Digital images of a diatom slide from the NMNH Botany collection (USNM 2703). **A)** Complete image of the slide (lower left) with circular coverslip outlined with a blue rectangle. The scanned rectangle is shown in red on the lower right. The main image shows specimens in the scanned area. B, **C)** Magnified images of two biraphid diatoms from within the scanned area in A, (B) a symmetric cf. *Geissleria* and (C) an asymmetric cf. *Cymbella*. Ra – Raphe, St – Striae, CN – Central nodule. Bar scales: A) 500 µm, (B) 25 µm, (C) 50 µm.

**Fig 6:**
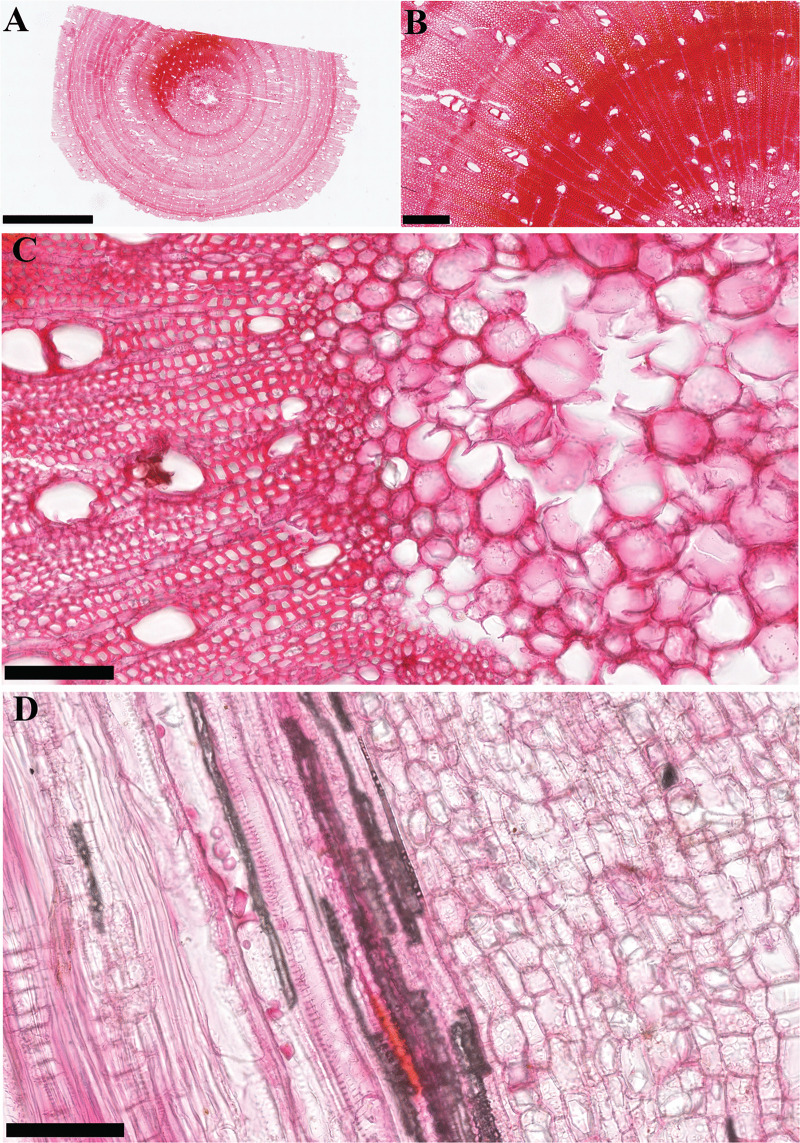
Digital images of modern wood vascular tissue from samples from the Instituto Humboldt, Colombia. (A, B, **C)** Transverse section. **(D)** Tangential section. Bar scales: (A) 2.5 mm, (B) 250 µm, (C, D) 100 µm.

**Fig 7:**
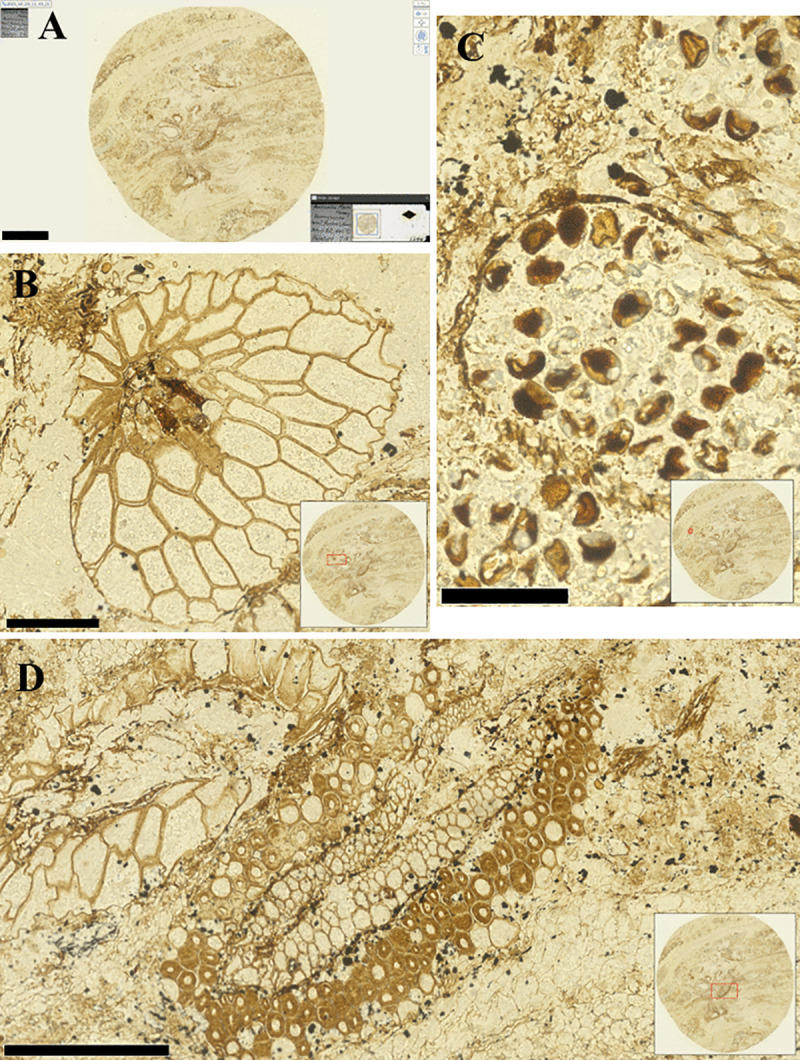
Digital images of the paratype of *Bowmanites moorei* Mamay (Mamay 1959) from the Pennsylvanian (~300 Ma) USNM P40935. **(A)** Image of the complete slide (lower right inset) and the mounted acetate peel. **(B)** Oblique transverse section of a sporangium (red box in inset photo shows position on peel). **(c)** Spores inside the sporangia (red box in inset photo shows position on peel). Bar scales: (A) 2.5 mm, (B) 250 µm, (C) 100 µm, (D) 500 µm.

**Fig 8:**
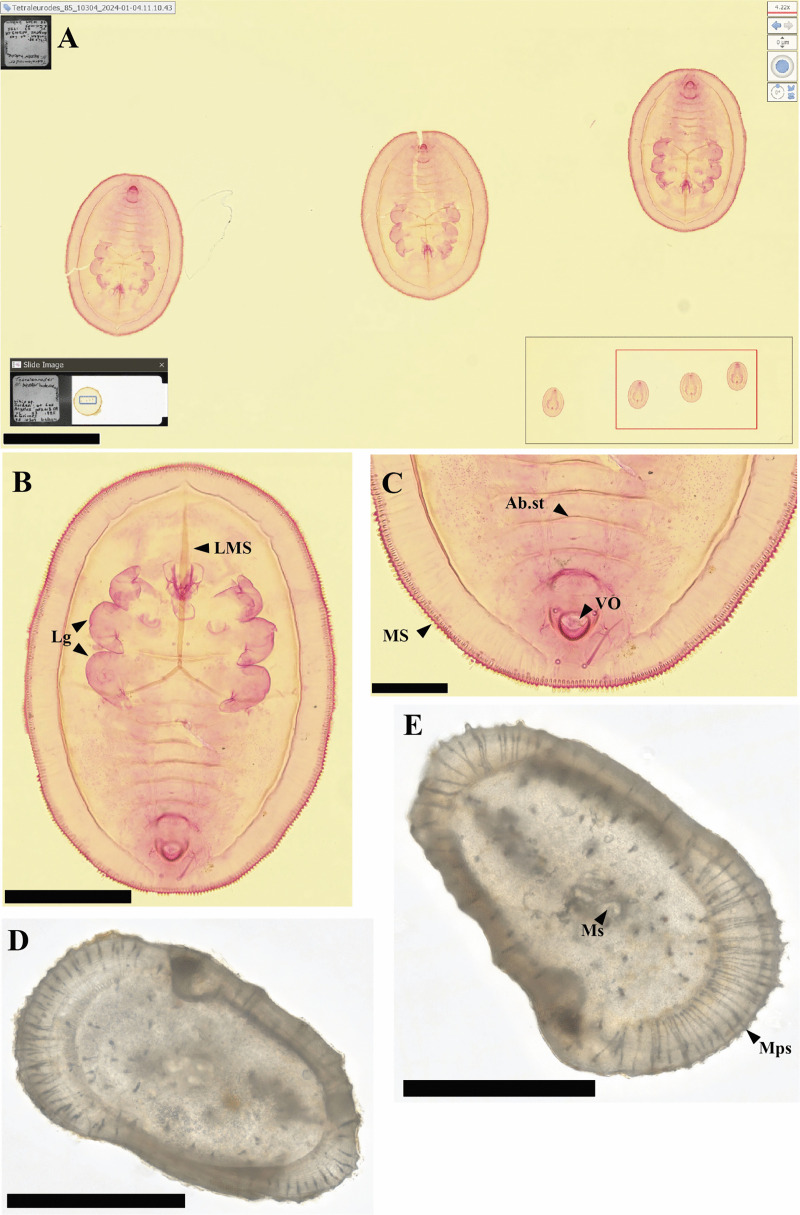
Digital images of: (A, B, C) the ventral surface of a nymph of genus *Trialeurodes* from California from the USDA entomology collection (USDA 10304). The inset image in the lower left of A shows the entire slide with a circular coverslip and the scanned region in a blue rectangle. The inset image on the lower right shows the scanned region and the enlarged central image indicated by the red rectangle. **(B)** Zoom in on a single nymph. LMS – Longitudinal, Lg – Legs. **(C)** Ab.st – Abdominal suture, MS – Marginal setae, VO – Vasiform orifice. **(D, E)** Fossils ostracods *Praephacorhabdotus pokornyi*, from the Late Cretaceous of Texas. **(E)** Ms – Muscle scar, Mps – Marginal pore canals. These two fossils are stored in the NMNH Paleobiology Ostracoda collections. Bar scales: (A, B, D, E) 250 µm, (C) 100 µm.

The latest generation of digital slide scanners provides a ground-breaking solution for imaging specimens mounted in microscope slides and stored in NHC, especially those showing signs of deterioration. Microscope slides imaged in the NZ slide scanner are captured at a high resolution (229 nm/pixel) and high speed (484 mm^2^ scanned in ~50 seconds). From the specimens selected as examples for this manuscript, the NZ scanning speed varied from 41 seconds to 18 minutes (Table 1). The fastest specimen was a single ostracod, which had a small, scanned area and 71 Z-stack layers (Table 1, Fig 8D), while the slowest sample was a fossil pollen slide with a larger scan area (20 x 20 mm), 25 Z-stack layers, and about 41,000 microfossils (Table 1, Fig 4). Most of the larger specimens, such as plant tissue, coal peels, and invertebrates scanned in 1–3 minutes (Table 1, Figs 6–8). For microfossil slides containing pollen, diatoms, and radiolarians scan times were longer (15–20 minutes) because large areas were captured with many planes of focus (Table 1, Figs 4, 5, S1). Although the scanning time for microfossil slides is relatively long, the benefits are large because many specimens become available for viewing, identification, measurement, and analysis (S1, S2 Videos).

Considering both the NZ scanning time (see *Timing of scanning* section) per slide and the required manual labor (5–15 minutes), which includes loading slides and setting the scanning profile, the total time required to scan 20 slides (the number of slides loaded into the NZ slide cassette) varies by sample type. For 20 palynological slides, using a 20 mm × 20 mm scanning area and a Z-stack of 25 focal planes, the total scanning time is approximately 6 hours. For 20 botanical or palaeobotanical slides, scanning time ranges from approximately 50 minutes to 1 hour. Scanning 20 ostracod slides requires about 30 minutes, whereas scanning 20 slides containing invertebrates, such as insects, typically takes between 1.5 and 2 hours.

The NDPI images of the selected specimens show the extent to which digital slide scanners can provide close approximations of the physical specimens (Figs 4-8, S1, S2 Fig). These images can also be annotated by including measures or ID names, which can then be exported for analysis. When zooming in on specific fields of view in the pollen, diatom, and radiolarian samples, different specimens can be detected, and differences in visible morphological characteristics facilitate the identification of the specimens (Figs 4, 5, S1 Fig). Some of the important morphological structures visible in the NDPI images that allow the identification of specimens are the number and types of apertures (pollen), number and arrangement of spicules (radiolarians), striae and raphe (diatoms), and arrangements of the wall (Figs 4, 5, S1). In plant tissue, different cells or parts of vascular systems can be identified, such as in wood tissue, where it is possible to identify various components of the secondary xylem, including fibers, parenchyma, and vessels (Fig 6), and in cuticles, different cells such as pavement epidermis and stomata (Fig S2). NDPI image files of coal ball peels also capture the cellular structures of plants, for example, the peel from an incomplete cone of the paratype of *Bowmanites moorei* shows sporangial structures, such as a sporophyll disc, some trilete spores, and other structures of its primary xylem (Fig 7).

Invertebrates imaged in NZ facilitate the detection and analysis of different parts of the organism and potential organs used for identification. In insects, several morphological characters are useful for identification, including mouthparts, reproductive organs, characteristics of the legs, antennae, and eyes (Figs 8a, 8b, 8c, S3). In fossil ostracod shells, some visible structures are details of the margin, ridges, and muscle scars (Figs 8d, 8e).

## Discussion

### Estimation of unknown diversity housed in NHC

Natural History Museums typically consider individual slides in slides collection as specimens when these contain biological or geological material. However, many of these slides contain hundreds to thousands of individual specimens that, with the advent of WSI, can now be tracked and identified individually [20,24,48]. This capability enables more accurate estimates of the diversity housed in NHC slide collections from NHC, as well as improved documentation, accessibility, and reuse of individual specimens mounted in a slide. To illustrate the magnitude of information that can be revealed by digitizing microscope slides, we estimated the number of specimens contained inthe 70,000 microscope slides of the DPC. For this purpose, we selected 78 slides and scanned two areas of 400 mm^2^ per slide. In one of the scanned areas, we selected a smaller annotated region of 2.89 mm^2^. The mean number of microfossils identified per annotated area was 111 (95% CI: 69–142), median = 63 (95% CI: 42–80), min = 16, max = 1302, SD = 165 (S2 Table). When extrapolated to the full coverslip area of approximately 1,600 mm^2^, this corresponds to an estimated mean of 61,574 fossils per slide (95% CI: 38,322–78,480) and a median of 34,879 (95% CI: 23,253–44,291). Based on this extrapolation, the 70,000 slides of the DPC at Smithsonian NMNH contain approximately 4.31 billion individual fossil specimens (95% CI: 2.68 bn – 5.49 bn).

For comparison, the entire collection of the Smithsonian NMNH had been estimated to comprise ~145 million specimens, and the sum of NHC at the 73 largest natural history museums in the world was estimated at 1.15 billion [19]. Clearly, those broader estimates do not reflect the vast number of microscopic fossils and other microscopic specimens housed on slides in the global NHC. Even for the Smithsonian NMNH, our estimates reported here do not include microscope slides from other collections, such as the marine microfossil slides housed in Paleobiology, the diatom and pollen collections in the Department of Botany (USNH), and many others. The global NHC is likely to be orders of magnitude larger and more scientifically valuable than currently realized once microscope slide collections are included.

We estimated the total time required to scan the 70,000 DPC slides and the associated data-storage needs. Scanning a single area of 400 mm^2^ with 25 focal planes spaced at 1µm intervals takes approximately 18 minutes. Most DPC slides contain either two cover slips or a single long cover slip, which is typically divided into two scan areas (left and right). Consequently, 10 slides can be scanned in about 6 hours, and 40 slides in one day. Based on these estimates, scanning the entire DPC collection would require roughly 4.8 years of continuous operation. Assuming an average file size of ~25GB per slide, the total storage requirement would be about 3.5 PB (Petabytes).

Although imaging the entire collection represents a substantial effort, the scientific return is even greater, enabling the investigation of major ecological and evolutionary questions in plants and fungi, and also providing critical data for modeling the effects of climate change on extinction, diversification, and changes in geographic distributions, among others [24]. Although the storage capacity required for a large microfossil collection is very large, it is likely that automated detection methods using artificial neural networks will soon be able to identify and copy only the parts of the original slide image that contain specimens of interest. This would allow the original images to be stored on tape backup and make it possible to share and collaborate using much smaller files containing images of one or a few specimens of interest.

### Three-dimensional data

One of the key advantages of slide scanning is the ability to acquire three-dimensional data (S1 Video). By capturing Z-Stack layers in bright-field microscopy, this system enables the detection of diagnostic morphological features that are not visible in a single focal plane (S1, S2 Videos). The resulting 3D-morphological datasets with the associated metadata facilitate quantitative analyses and support high-throughput applications, including machine learning.

Recent studies have demonstrated that images obtained with slide-scanner systems provide new metadata, key to applying high-throughput machine learning approaches to advance research related to the detection, identification, and classification of palynological and paleobotanical material. One of the first studies using a microscope slide scanner in palynology was Tcheng et al. [22], in which they scanned 34 pollen samples to compare the pollen morphology of two species of spruce, *Picea mariana* and *Picea glauca*, using an open-source visual recognition software known as ARLO (Automated Recognition with Layered Optimization). Punyasena et al. [23] developed an automatic workflow for pollen analysis from scanned slides using convolutional neural networks (CNN). Studying tropical specimens from Panama, they achieved over 80% accuracy in identifying the 25 most frequent taxa. More recently, Martinsen et al. [19] introduced a pipeline for the identification and classification of microfossil species using a self-supervised computer vision model. Analyzing 100,000 images, their method successfully extracted morphological features for species identification, grouping, and counting. These studies highlight the importance of three-dimensional data in improving model performance, especially in Palynology. The added depth provided by Z-stack imaging enables the capture of more detailed morphological traits, which significantly enhances the effectiveness of computer vision models for taxonomic analysis.

Similar applications can be developed for other types of mounted-slide material, such as plant, fungal, and animal tissue, or small invertebrates digitized with slide-scanners, with the added advantage of capturing three-dimensional structures. Recent studies have developed or applied approaches that integrate data visualization and image analysis while optimizing labor investment for two-dimensional images. For plant tissue analysis, Lloyd et al [49] developed the CuticleTrace toolkit, a suite of Fiji and R-based functions that automate the tracing and measurement of epidermal cells. They demonstrated that this toolkit can process leaf cuticle images rapidly, producing expert-level measurements of epidermal cells, generating larger datasets in a shorter time than manual tracing [49]. Other similar tools include LeafNet [50] and StomataCounter [51]. High-throughput methods exist for identifying and analyzing invertebrates larger than typical microscope slide specimens [52,53]. While these methods have not yet been applied to slide-mounted invertebrates, their underlying principles could be adapted for smaller specimens.

### Replicability, accessibility, and collaboration

One of the primary challenges in the analysis of microscope specimens, particularly in fields such as palynology and micropaleontology, is the difficulty in verifying taxonomic identifications and reproducing study results [24,49,50]. This issue arises from a combination of factors, including variability in microscopy techniques, differences in analyst expertise, and a lack of standardization [49,50]. A full replication of specimen identifications is hindered by the fact that the microscope coordinates of only a small number of identified specimens are annotated, and the physical exchange of glass slides between institutions can be difficult due to logistical and transportation constraints [24].

Digital slide scanning technology offers a promising solution to these limitations. Slide scanners enable the high-resolution digitization of entire microscope slides, allowing the sharing of images, specimen data, and associated annotations among researchers and institutions. This digital workflow enhances the reproducibility of analyses and improves the accuracy of taxonomic identifications through collaborative verification [24]. It also enables reimaging, identification review, and new morphological analysis of historical specimens previously studied.

As examples, we imaged the holotype and allotype of *Ctenidiosomus perpexus* (S3 Fig), described by Tipton and Machado-Allison [51], as well as the paratype of *Bowmanites moorei* (Fig 7), described by Mamay [52]. The resolution and detail obtained in these images allow re-evaluation of previously described morphological features, including internal structures and organs. Importantly, this approach facilitates access to specimens without the need to handle or physically access the original slides. An additional advantage of digital slide files is the ability to incorporate measurements and annotations, as well as to use these data in high-throughput deep-learning and machine-learning analyses [53–59].

In palynology, the positions of microfossils have been historically recorded using microscope coordinates, or an England Finder (EF), an indexed microscope slide used to document and relocate areas of interest. Slide scanners, such as the NZ, incorporate an internal coordinate system in which every location within the scanned area has a unique X and Y coordinate point (S4, S5 Figs). This system facilitates tracking and recording of individual specimens within each slide and enables the translation of EF positions previously recorded manually into the WSI framework. When combined with OMERO, this coordinate system supports the assignment of unique identifiers to individual specimens within a single slide [20,24], making it possible to track and catalog hundreds to thousands of specimens per slide. Thus, digitization significantly expands opportunities for remote collaboration among scientists worldwide [2,24]. It facilitates access to diverse collections and specimens, even when physical mailing is impractical or when researchers are unable to travel to review samples in person. Collectively, these advantages help to broaden access to critical scientific resources and foster broad-based and widespread participation in research.

## Conclusions

The estimated 4.3 billion individual microfossil specimens from the DPC housed at the Smithsonian NMNH exemplify the vast, largely unused scientific potential rooted in slide collections within natural history collections globally. These specimens represent an extraordinary, high-resolution record of Earth’s biological and environmental history, but their full research value remains inaccessible without large-scale digitization.

Digitization of microscope slides is, therefore, not only a matter of preservation but also a prerequisite for discovery. The combination of high-resolution digital imaging and emerging machine learning approaches provides an unprecedented opportunity to extract, quantify, and analyze morphological and ecological information at scales previously unimaginable. Leveraging these technologies could transform how natural history collections are explored, enabling automated identification, accelerating taxonomic work, and revealing hidden patterns in global biodiversity and evolution. Unlocking the information contained in billions of digitized specimens will expand the reach and relevance of the world’s natural history collections, turning them into dynamic, data-rich resources for future scientific innovation.

## Supporting information

S1 Fig Digital images of radiolarians from the ODP core 113-689B sample slide 34–40 cm from the middle Eocene from Antarctica. This sample is from the ODP core slide samples from the NMNH Paleobiology collections. Bar scales: (A) 500 µm, (B) 250 µm, (C) 100 µm.
(TIF)

S2 Fig Digital images of: (A, B, C) fossil leaf cuticles from the Paleogene (55–57 Ma) from the USA; and (E, F) *Prototaxites* tissue, a fossil fungus from the Devonian (~407 Ma) from Canada. Bar scales: (A) 2.5 mm, (C) 1 mm, (D, F) 250 µm, (B) 50 µm.
(TIF)

S3 Fig Digital images of: (A, B, C, D, E, F) two beetles from the family Ptiliidae, from the Field Museum Entomology collection, (A, B) FM 46745 and (C, D, E, F) FM 66–245; and (G, H, I, J) two fleas from the NMNH Entomology collection, corresponding to the (I, J) holotype and (G, H) allotype of *Ctenidiosomus perpexsus.* Bar scales: (C, D, F) 100 µm, (A, B, J) 250 µm, (E, G) 500µm, (H, I) 1 mm.
(TIF)

S4 Fig Graphic representation of the area that can be scanned in the NZ and its coordinate system. A) Shows the NZ scan area (gray) with five circles representing different annotations, blue circles are located in the corners of the NZ scan area, and the red circle is located in the center of the area representing the coordinate 0,0. B) Represent different scenarios of where the five annotations will be located based on the position of the scanned area (dark orange).
(TIF)

S5 Fig Three scanned slides in which the scanned area (blue box of the inset image, located in the bottom corner). The coordinates of the five annotations in each slide are the same across the slides, corner annotations (blue circles) and the middle annotation, which is 0,0 (red circle). A, C) Only three annotations fell into the scanned area. B) All annotations fell in the scanned area.
(TIF)

S1 Table Collection of information on the specimen selected to show variation in digitization using the NZ slide scanner. (PDF)

S2 Table Number of microfossils identified in a delimited area of 2.89 mm^2^, in 66 pollen slides from the USGS fossil pollen collection. (PDF)

S1 Video Zooming into the scanned slide of fossil pollen from the Miocene (11−5 Ma) from Alaska (sample D8401), part of the DPC housed at NMNH Paleobiology. (MP4)

S2 Video Visualization across the Z-plane to visualize different morphological characteristics and body parts of a beetles from the family Ptiliidae. (MP4)

S1 File Step-by-step protocol to use a NanoZoomer S20 Digital slide-scanner to digitize microscope slide-based Natural History Collections. (PDF)
