# Supplementary figures and images for: Digitizing microscope slide-based natural history collections: A protocol using slide scanner technology
Source: PLoS One. 2026 Apr 24;21(4):e0346139. doi: 10.1371/journal.pone.0346139 (PMC13108749; doi:10.1371/journal.pone.0346139)

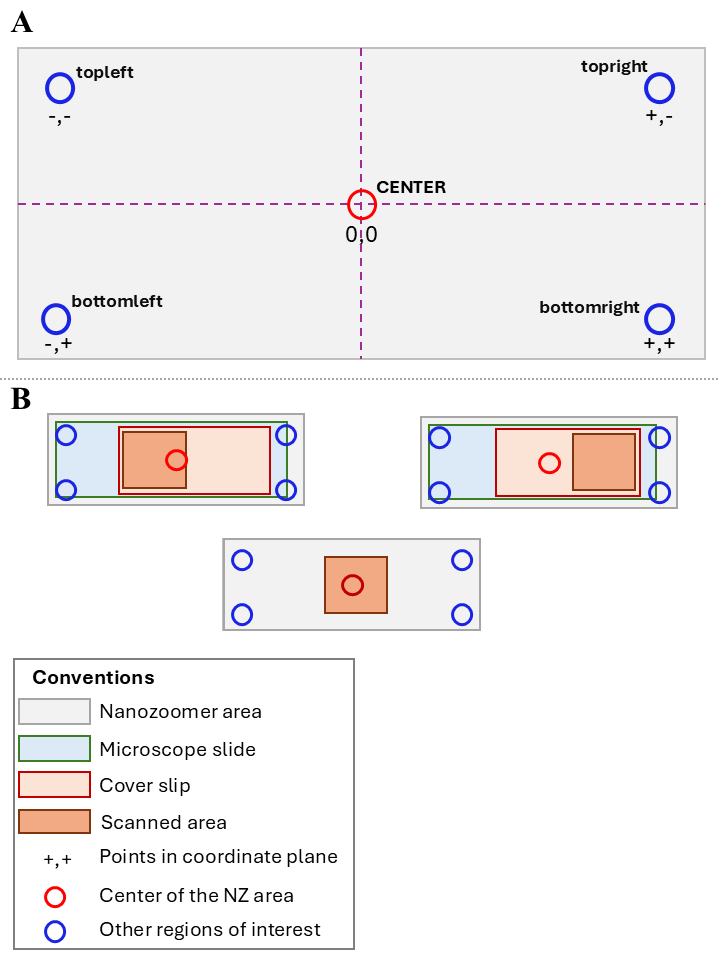

Supplement: S4 Fig — A) Shows the NZ scan area (gray) with five circles representing different annotations, blue circles are located in the corners of the NZ scan area, and the red circle is located in the center of the area representing the coordinate 0,0. B) Represent different scenarios of where the five annotations will be located based on the position of the scanned area (dark orange). (TIF) [file pone.0346139.s004.tif]

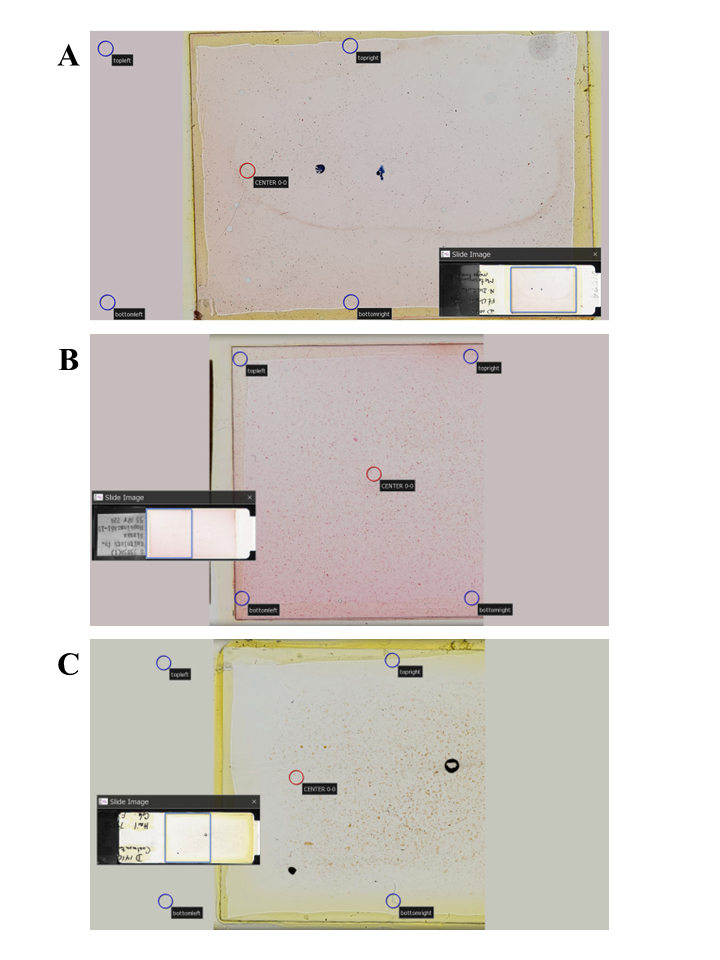

Supplement: S5 Fig — The coordinates of the five annotations in each slide are the same across the slides, corner annotations (blue circles) and the middle annotation, which is 0,0 (red circle). A, C) Only three annotations fell into the scanned area. B) All annotations fell in the scanned area. (TIF) [file pone.0346139.s005.tif]
