# Supplementary material for: Digitizing microscope slide-based natural history collections: A protocol using slide scanner technology
Source: PLoS One. 2026 Apr 24;21(4):e0346139. doi: 10.1371/journal.pone.0346139 (PMC13108749; doi:10.1371/journal.pone.0346139)
Supplement: S1 Table — (PDF) [file pone.0346139.s006.pdf]

S1 Table. Collection of information on the specimens selected to show variation in digitization using the NZ slide scanner

Specimen type	Modern /Fossil	specimen/ Collection ID	Museum	Collection	Country	File Name	Specimen provided by
1.Pollen	Fossil	D8401	NMNH	Paleobotany	USA	1_D8401-A_L_2024_01_18_13_05_54_Alaska	Ingrid Romero
2.Diatoms	Modern	2703	NMNH	Diatoms	USA	2_2703_USGS_2023-11-06 10_16_13	Leslie Brothers
3.Radiolarians	Fossil	113-689B-13H-02	NMNH	Radiolarians	USA	3_113-689B_13h-02_34-40_L_2025_09_16_13_15_27	Brian Huber
4.Fungi	Fossil	Prototaxites	NMNH	Paleobotany		4_Prototaxites_fossil_fungi_NMNH	Jonatan Wingerath
5.Cuticle	Fossil	Cuticles	NMNH	Paleobotany	USA	5_Cuticle_2023-12-13 13.27.36	Jon Milligan
6.Coal ball peel	Fossils	USNM P 40935	NMNH	Paleobotany	USA	6_40935_Bowmanites_moorei	Ingrid Romero
7.Wood tissue	Modern	8	Instituto Humboldt	Botany	Colombia	7_Colorados_8_2024_05_15_14_45_00	Diana Perez
8.Ostracod	Fossil	Ostracod A	NMNH	Ostracoda	USA	8_Slide_ostracod_A_specimen_fossil_NMNH	Gene Hunt
9.Ostracod	Fossil	Ostracod B	NMNH	Ostracoda	USA	9_Slide_ostracod_B_specimen_fossil_NMNH	Gene Hunt
10.Flea	Modern	3885	NMNH	Entomology	Venezuela	10_Ctenidiosomus_perpexsus_Holotype_2025_09_08_12_31_29	Torsten Dikow
11.Flea	Modern	3885	NMNH	Entomology	Venezuela	11_Ctenidiosomus_perpexsus_Allotype_2025_09_08_12_43_17	Torsten Dikow
12.White flies	Modern	10304	USDA	Entomology	USA	12_Tetraleurodes_85_10304_2024-01-04 11.10.43	Ian Stocks
13.Coleoptera	Modern	FM(HD)#66-24	Field Museum	Coleoptera	USA	13_Ptiliidae_FM_46745_2025_09_08_12_49_22	Bruno de Medeiros
14.Coleoptera	Modern	FM(HD)#B745	Field Museum	Coleoptera	USA	14_Ptiliidae_FM_66-245_2025_09_08_12_38_40	Bruno de Medeiros
