# Supplementary material for: Digitizing microscope slide-based natural history collections: A protocol using slide scanner technology
Source: PLoS One. 2026 Apr 24;21(4):e0346139. doi: 10.1371/journal.pone.0346139 (PMC13108749; doi:10.1371/journal.pone.0346139)
Supplement: S2 Table — (PDF) [file pone.0346139.s007.pdf]

S2 Table. Number of microfossils identified in a delimitated area of 2.89 mm^2, in 66 pollen slides from the USGS fossil pollen collection.

File_name	USGS_sample_ID	Palynomorps detected in an area 2.89 mm^2
D1821_1_L_2024_01_22_16_22_31_New_Mex	1821	47
D1821_1_R_2024_01_23_15_01_49_New_Mex	1821	35
D1910_2024_02_29_12_46_52_Wyoming	1910	30
D1911_1_2024_02_29_18_10_06_Wyoming	1911	85
D3094_1_L_2024_02_20_11_32_26_Colorado	3094	147
D3094-1_R_2024_02_20_17_15_37_Colorado	3094	171
D3202_C_L_2024_02_20_12_31_51_Louisiana	3202	196
D3202-D-1_R_2024_02_21_13_01_51_Louisiana	3202	84
D3202_C_R_2024_02_20_18_12_34_Kentucky	3202	216
D3266-1_L_2024_02_01_13_49_20_Mississippi	3266	62
D3268_A-2_L_2024_02_01_12_52_49_Alabama	3268	25
D3283-2_2024_02_06_15_37_28_Kentucky	3283	66
D3408_1_R_2024_02_20_19_10_35_Texas	348	351
D3408-1_L_2024_02_20_13_47_16_Texas	3408	474
D3516-A_1_L_2024_02_20_12_51_02_Montana	3516	29
D3516-A_1_R_2024_02_20_18_31_40_Montana	3516	31
D3516-B_2_L_2024_02_20_14_24_29_Montana	3516	44
D4200_2_2024_02_06_12_29_47_Wyoming	4200	28
D4354_3_R_2024_02_05_12_39_12_Arkansas	4354	51
D4220_A_L_2024_02_06_11_51_42_Maryland	4220	61
D5151-B-1_L_2024_02_02_15_31_05_Tennessee	5151	337
D5406_1_L_2024_02_09_10_25_26_Colorado	5406	61
D5410_1_L_2024_02_09_10_06_56_Utah	5410	39
D5410_1_R_2024_02_0911_03_13_Utah	5410	30
D5542_1_L_2024_02_06_10_55_26_Colorado	5542	75
D5609_L_2024_03_01_11_15_57_Alaska	5609	89
D5609_R_2024_03_01_15_10_09_Alaska	5609	71
D5825_L_2024_02_06_10_36_15_Utah	5825	30
D6627-A-2_R_2024_02_02_17_42_28_Arkansas	6627	137
D8401-A_L_2024_01_18_13_05_54_Alaska	8401	297
USNM_792877_D1727_L_2024_03_14_10_25_59_Washington	1727	166
D9151-A-2_L_2024_02_02_16_08_52_Texas	9151	1302
USNMPAL_792673_D1166_A_L_2023_11_09_12_47_47_Wyoming	1166	222
USNMPAL_792673_D1166_A_R_2023_11_09_15_17_21_Wyoming	1166	117
USNMPAL_792715_D1298_L_2024_01_25_14_55_02_Wyoming	1298	41

USNMPAL_792717_D1350_L_2024_02_20_15_21_31_Oregon	1350	131
USNMPAL_792719_D1357_2023_11_09_10_41_27_Colorado	1357	195
USNMPAL_792723_D1366-D_2024_09_19_09_27_16_Maryland	1366	150
USNMPAL_792724_D1367_A_2024_01_25_15_32_32_Maryland	1367	33
USNMPAL_792725_D1367-B_2024_09_19_09_45_18_S_Dakota	1367	62
USNMPAL_792733_D1388_L_2024_01_22_11_09_00_S_Dakota	1388	82
USNMPAL_792738_D1392_A_R_2024_01_25_13_58_00_Wyoming	1392	67
USNMPAL_792851_D1644_2024_01_26_11_12_23_California	1644	37
USNMPAL_792852_D1667-A_L_2024_02_20_14_05_57_Kentucky	1667	103
USNMPAL_792854_D1668_L_2024_01_25_16_38_58_Kentucky	1668	64
USNMPAL_792930_D1825_L_2023_11_07_13_36_14_image1_Kentucky	1825	98
USNMPAL_792954_D1890-4_L_2024_09_19_11_55_30_Kentucky	1890	41
USNMPAL_793077_D3163-A_2024_07_29_10_45_13_Arkansas	3163	389
USNMPAL_793367_D3702_R_2024_02_05_12_01_01_Tennessee	3702	109
USNMPAL_793471_D3915_L_2024_01_22_10_22_34_Alaska	3915	113
USNMPAL_793472_D3916_R_2023_11_20_17_04_43_Alaska	3916	82
C_418080_W_Nassichuk_L_2025_01_14_19_14_58_Alaska	418080	165
C_418058_W_Nassichuk_R_2025_01_21_14_59_16_Alaska	418058	144
C_418081_W_Nassichuk_R_2025_01_21_12_58_10_Alaska	418081	126
C_418070_W_Nassichuk_R_2025_01_21_14_17_12_Alaska	418070	27
C_418107_W_Nassichuk_R_2025_01_21_11_16_25_Alaska	418107	107
C_418114_W_Nassichuk_L_2025_01_14_16_15_48_Alaska	418114	260
D3869-1_R_2024_02_20_17_53_41_Wyoming	3869	42
D6627-A-2_L_2024_02_02_12_53_43_Arkansas	6627_A	79
USNMPAL_793367_D3702_L_2024_02_02_15_49_54_Tennessee	3702	46
USNMPAL_792852_D1667-A_R_2024_02_2019_29_55_Kentucky.ndpi	1667	169
D5964-1_L_2024_02_06_10_17_07_Wyoming	5964	28
D3407-2_L_2024_02_20_13_28_48_Texas	3407	114
USNMPAL_792924_D1820_2024-01-23_13_40_57_N_Mexico	1820	49
Giraffe_26_3_146_L_Giraffe_2025_01_13_16_06_02	26_3_146	30
C_418109_W_Nassichuk_R_2025_01_21_10_57_19	418109	56
