# Supplementary material for: Digitizing microscope slide-based natural history collections: A protocol using slide scanner technology
Source: PLoS One. 2026 Apr 24;21(4):e0346139. doi: 10.1371/journal.pone.0346139 (PMC13108749; doi:10.1371/journal.pone.0346139)
Supplement: S1 File — (PDF) [file pone.0346139.s010.pdf]

Step-by-step protocol to use a NanoZoomer S20 Digital slide-scanner to digitize microscope slide-based Natural History Collections

RESERVED DOI:
10.17504/protocols.io.bp2l6ex61gqe/v1

Ingrid Romero[1,2], Scott Wing[1], Carlos Jaramillo[2]

[1]Smithsonian National Museum of Natural History; [2]Smithsonian Tropical Research Institute

incaromero

Protocol Info: Ingrid Romero, Scott Wing, Carlos Jaramillo . Step-by-step protocol to use a NanoZoomer S20 Digital slide-scanner to digitize microscope slide-based Natural History Collections. **protocols.io** **https://protocols.io/view/step-by-step-protocol-to-use-a-nanozoomer-s20-digi-hmjkb44kx**

Manuscript citation:
Romero I. C., Wing S., Jaramillo C., Little H., Punyasena S. W, White A. E. Digitization microscope slide-based Natural History Collections: A protocol using slide scanner technology. PLOS One DOI: 10.1371/journal.pone.0346139

Created: January 19, 2026

Last Modified: March 31, 2026

Protocol Integer ID: 238924

Keywords: MIcroscope slide, natural history collection, botany, paleontology, palynology, entomology

Funders Acknowledgements:
Smithsonian Climate Change Fellowship
Grant ID: Our Shared Future: Life on a Sustainable Planet
SmithsonianLife on a Sustainable Planet Pathfinder Grant
Grant ID: Effects of extreme global warming on the distribution of terrestrial plants
Smithsonian Institution
Grant ID: Office of the Undersecretary for Science
National Museum of Natural History
Grant ID: Office of the Associated Director for Science and Chief Scientist
The Bill and Dianne Ryan
Grant ID: Family

Abstract

We present an optimized protocol for digitizing diverse microscope slide specimens using a NanoZoomer S20 slide scanner (NZ). We provide specimen-specific recommendations for scanning parameters, including scan area, focal points, Z-stack configuration, and file management workflows. After digitazing over a 1,000 slides from the Smithsonian natural history collection (NHC), containing insects, tissue material from plant, fungal, and animal, and microspecimes (ex: pollen, diatoms, and radiolarians). The total scanning time of individual slides varied from 41 seconds for small invertebrates to 18 minutes for palynological samples, with final compressed file sizes of 0.15 to 28 GB. This protocol provides practical guidance for institutions looking to digitize slide-based collections to preserve and unlock their full research potential. It also propose a solution to preserve natural history collections that are suceptible to deterioration.

Guidelines

This section includes recommendations and important information to consider when scanning different types of specimens mounted on microscope slides.

Scanning parameters: optimization by specimen type

When configuring the imaging profile in NZ, it is important to define several parameters, including bjectives, scan area, split tissue setting, number and positions of focal points, and the number and spacing of Z-stack layers. The parameter settings depend on the specimen type and size.

Objectives: The NZ system uses a 20X objective with a precision optical coupler that allows scanning in two modes, 20X with a 0.46 µm/pixel resolution and 40X with 0.23 µm/pixel resolution. This feature has the advantage that the time of scanning in both modes is similar (i.e, scanning speed of a 15 × 15 µm area will take ~30s in 20X and 40X).

Specimens such as insects, wood tissue, and coal ball peels can be imaged using both modes, 20X and 40X, while 40X is preferred for microfossils, such as pollen, diatoms, and radiolarians.

Scan area: The size of the selected scan area depends on the type of sample or specimen being imaged.

For samples with microfossil specimens (pollen, spores, dinoflagellates, radiolarians, including diatoms and phytoliths), which have hundreds to thousands of individual specimens per slide, we recommend selecting one or two scan areas of approximately 20 mm x 20 mm, depending on the cover slip size. This is generally sufficient to image most specimens, while keeping file sizes manageable for analysis ($\leq$ 30Gb), facilitating storage, management, and sharing of files among collaborators.

For larger specimens (>0.5 µm) such as cuticles, wood samples, fungi tissue, coal ball peels, and small invertebrates (e.g., ostracods and insects), the size of the scan area varies, based on the size and/or number of specimens present on the slide. We recommend avoiding scanning the edges of the cover slip as this can interfere with the scanner's ability to detect and focus on the specimen.

Focus points: The focal points are initially set automatically when defining the profile settings. However, even when a specific number is selected, the NZ may automatically adjust the number of focus points based on material detection, and in those cases, these points can be manually modified or moved.

For microfossil slides with 400 mm^2 selected scan areas, we recommend five focal points per subdivided scan area (see *Split tissue*); for larger scan areas, nine points are recommended. However, autofocus success depends critically on the method of slide preparation. Automatically placed focal points typically produced sharp images if the microfossils were applied to the coverslip, which was then inverted and affixed to the slide with mounting medium of uniform thickness.

Manual focal point adjustment is commonly required for historical collections, where the mounting medium shows signs of degradation, such as coloration, or when the material was mixed with mounting medium and then applied to the slides. In these cases, the scanner's automatic focal points often failed to detect material or lost focus because specimens were distributed unevenly across focal planes, and/or the mounting medium had deteriorated (dried and shattered), and the coverslip had deformed.

Manual focus is also recommended for slides in which the mounting medium has shattered, but the microfossils are preserved in good condition and can provide important information for biostratigraphy, or ecological and evolutionary inferences.

For larger specimens (>0.5 µm), the optimal number of focal points will vary based on the size of the specimen. For specimens that occupy the entire cover slip, using the "split tissue" function (see Steps section) is critical, as well as assigning five or nine focal points per subregion. For smaller fragments, such as cuticles, five points are generally sufficient, but the number may be adjusted according to the scan area.

Split tissue: For microfossils, as well as larger specimens, in which the scan area is larger than 10 mm x 10 mm, we recommend using the automated split function to divide the scanned area into several equal-sized subareas to increase the likelihood that most of the material will be in focus.
For slides with multiple large fragments or specimens (>0.5 µm), we recommend manually drawing scan areas around each specimen, rather than relying solely on automatic subregion detections (e.g., invertebrates and cuticle fragments). This allows the size of the scan area to vary based on the size of each specimen/fragment, improving focus.
Z-Stack: The Z-stack settings (number of layers and spacing between them) vary based on the specimen size and the details of morphological information that can be captured in the Z-space (*see more in the associated manuscript*). It is important to highlight that the number of layers selected is always an odd number because the middle layer is the central focal plane, which is set using the focus points, then the layers above (+) and below (-) are numbered accordingly. For example, if 15 layers are scanned, the microscope will image the middle layer plus seven above (layers +1 to +7) and seven below (layers -1 to -7).
For microfossils, the optimal Z-stack configuration depends on the depth of individual microfossils and how they are distributed along the Z-axis. For palynological slides (with pollen, diatoms, and radiolarians) from NHC, the Z-stack settings recomended is between 25 and 30 layers at 1 µm intervals because has proven effective in capturing not only the three-dimensional structure of individual microfossils but also most of the microfossils in the slide. For other slides in which all specimens are in a similar Z-plane because the concentrate was dried onto the underside of the cover slip before mounting, a Z-stack comprising 15 to 21 layers at 1 µm intervals may be sufficient to capture the full depth of the material along the Z-axis.
For larger specimens (>0.5 µm)**,** the number of z-stack layers varies with the size of the specimen. Most samples of leaf cuticles, wood and fungi sections, and coal ball peels can be scanned with three to five planes spaced three to five µm apart on the Z-axis. This produces focused images in spite of undulations in the specimens. For small invertebrates (e.g., fleas, beetles, and ostracods), the focal plane layers can vary from 31 to 81, according to the depth of the specimens. The intervals between layers can vary within 3 to 4 µm intervals for insects, while for ostracods and smaller insects, such as white flies, 2 µm intervals between layers capture more morphological details.
Timing of scanning
The NZ can scan an area of 15 mm x 15 mm in approximately 30s, this in both 20X and 40X modes. The final NDPI file always records the total scanning time, as well as the timing of the three processes included within it, which are focusing, scanning, and writing. *Focus time* refers to the period during which the NZ reviews each focal point to locate the material to be scanned and to define the middle focal plane. This time depends on the total number of focal points per slide. *Scanning time* corresponds to the time the NZ spends scanning a single slide and depends on the selected scan area and the number of focal planes. *Writing time* refers to the period during which the software generates the final NDPI file.
This time does not include manual operation of the scanner, which is defined by the time required to load and unload slides, set the scanning profile, and replace slides. This manual time varies from approximately 5 to 20 minutes. This includes 5 to 10 minutes uploading the slides, setting the scanning profile, and reviewing the profile settings in each sample, especially the scanning area and focal points. The other additional time includes unloading the slides and closing the system, or uploading another set of slides and restarting the scanning process.
It is important to highlight that once the settings for the first slide are finalized, the scanning can begin, while the parameters of the remaining slides are reviewed and adjusted. Therefore, this manual effort does not delay the

scanning process, as it can be performed in parallel with scanning, and only the time of uploading and unloading the slides can be added to the total scanning time provided by the NZ.

Troubleshooting

Safety warnings

Slide condition

- We do not recommend scanning barren microfossil slides, slides with very few specimens, or slides in which the mounting medium is shattered and the microfossils are destroyed, because the information preserved is not likely to justify the time, effort, and file storage space. We do recommend recording the reason a given slide was not scanned.
- The scanner's automatic focal points often failed to detect material or lost focus when specimens were distributed unevenly across focal planes, and/or the mounting medium had deteriorated (dried and shattered), and the coverslip had deformed. In these cases, setting the focal points manually is recommended.
- In cases where the mounting medium has shattered, but the microfossils are preserved in good condition and can provide important information for biostratigraphy, or ecological and evolutionary inferences, we recommend scanning the slide. If there is trouble with the automatic focus, setting the focal points manually is also recommended.

Ethics statement

No permits were required for the described protocol, which complied with all relevant regulations

Before start

Before starting scanning, slides should be reviewed to ensure scanner compatibility and prevent slide breakage. It is also important to review the slide to exclude barren slides or slides in which the specimens are too deteriorated or broken. Additionally, it is necessary to clean the whole slide before inserting it in the scanner.

Slides compatibility with the slide scanner

1. The Nanozoomer S20 (NZ) only accepts standard pathology slides (75 × 25 × 1 mm). However, other slide scanners, such as Zeiss Axioscan and Olympus VS200, have features that allow the scanning of samples mounted in slides with different dimensions (e.g., 1×3″, 2×3″, 4×3″).
2. The cover slip and label must be affixed to the same side of the slide, with nothing attached to the back of the slide or extending beyond the edges of the slide.
3. The label must lie completely flat.
4. The NZ's autofocus works best when the cover slip lies level to the slide surface.

Selection of slides to scan

1. To rapidly assess whether a slide is suitable for digitization, it is important to verify that the mounting medium is not deteriorated or severely degraded. Additionally, when viewed against the light, the slide should exhibit a cloudy appearance, which indicates particulate organic matter. This, combined with a brief inspection under an optical microscope, in which if the slides present multiple specimens in at least five fields of view, makes the slide good for scanning.

Naming image files

1. Naming protocols should be established before any large-scale digitization effort.
   - If the slides have 1D or 2D barcodes, the NZ has linear image sensors that can read the codes to name the files during the scanning process, ensuring the specimens' unique names and collection identifiers are captured, and reducing errors in transcribing specimen data.
   - If the slides do not have barcodes, a file naming format needs to be established beforehand. Then, create a .txt or .csv file with a list of sample names to import before starting the scanning. In the file, do not add titles. List the names in the order the slides will be put in the NZ.

1. Start-up and insertion of slides in the NanoZoomer

1 Turn on the NanoZoomer (NZ).

2 Turn on the NZAcquire software.

3 Click on “Cassette Access”.

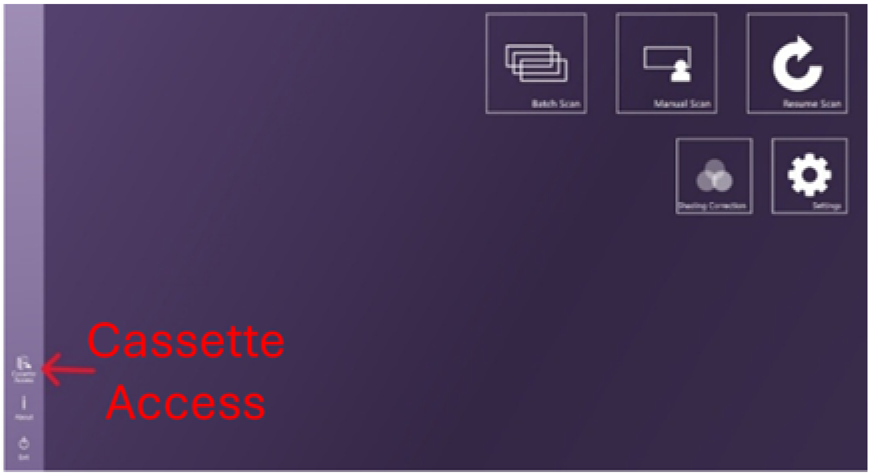

4 When ready, NZ will make a noise indicating the door can be opened, and the cassette can be extracted. To extract the cassette, tilt it outward at approximately 30° before removing it.

5 Insert the slides into the cassette. Coverslips should face up and labels should face outward from the cassette. This steps takes approximately five minutes if the slides have been previously selected and organized.

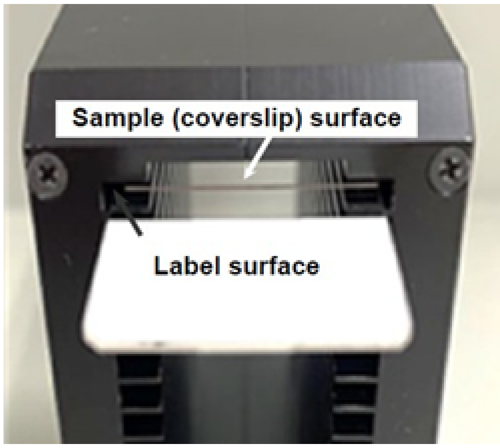

6 Insert the cassette into the NZ and close the door (left, middle). Then, in the NZAcquire software, **click OK**. Positions with properly placed slides will appear in white (right). Empty positions will be unlit. Misaligned or faulty slides will appear in red.

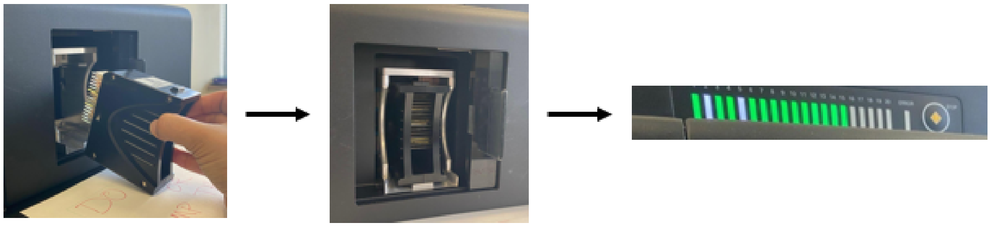

Left: Shows how to put the cassette in. Middle: Shows the cassette inside the NZ. Right: Shows the NZ indicating the positions of the slides placed in the cassette. The green light is after scanning.

7 Select “**Batch Scan**”. NZ has two modes, “Full Automatic” and “Semi Automatic”. If “Batch Type” is set to “Fully Automatic”, click and change it to “Semi Automatic”.
NOTE: *The **fully automatic mode** is suitable for slide collections in which labels and cover slips are placed in the exact same position across all slides, but Natural History Collections (NHC) are typically too heterogeneous.*
***Semiautomatic mode** is recommended for heterogeneous samples, such as those in NHC, because it allows manual review and adjustment of the label and scan area for each slide, when necessary, and before the scanning begins.*

2. Setting the scan parameters and profile

8 Note before starting this section: Setting the profile and defining scanning parameter can take about 5 minutes.
Capture a macro image by clicking the camera icon “Taking a slide image”.

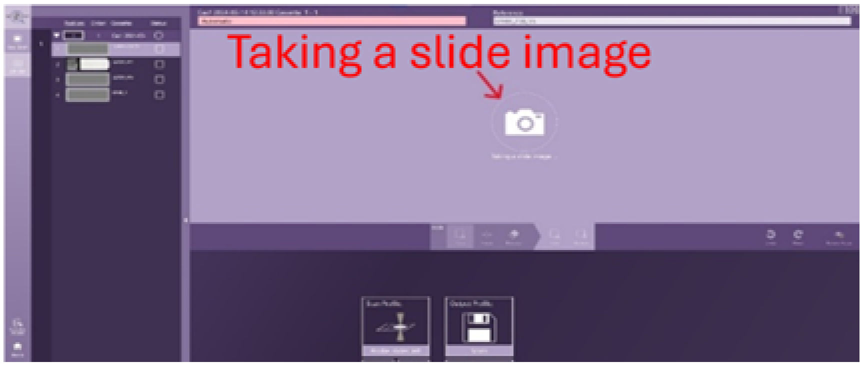

9 Click on "List View".

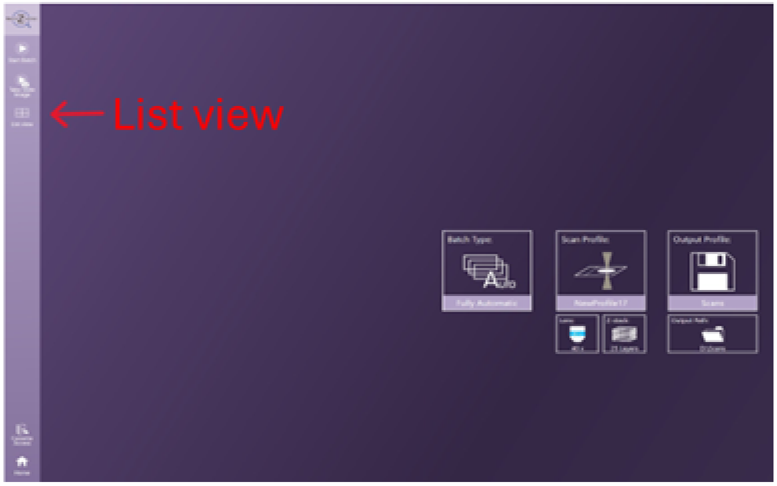

Note: It is not necessary to wait until all macro-images have been taken to go to "List view"

10 Click in the white triangle (yellow arrow) if it is not facing down, to display the samples in the cassette. Check the small white box (red arrow) under the cassette to modify sample information before scanning.

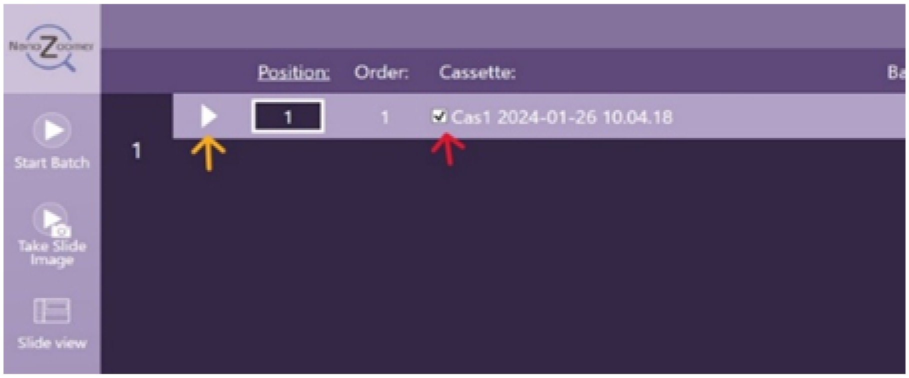

The white triangle displays the information in the cassette, such as the number of slides, the name of each sample, and the profile settings.

11 In the bottom-right of the screen, click on "Cassette information".

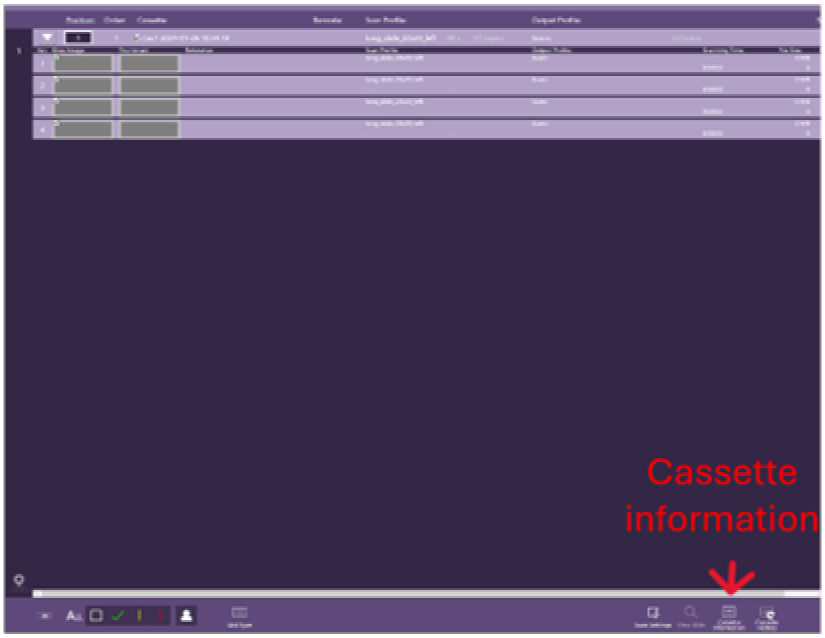

12 Select “Scan Profile”. This section allows to adjust settings for the batch of samples, including barcode area, scan area, lens magnification, and Z-stack.

13 Choose one of the existing profiles (red arrow) or create a new one. To edit an existing profile, click on **Edit** (blue arrow). To create a new profile, click on **New** (yellow arrow). Here, you can set the default scanning parameters for your samples. Review the *Guidelines and Warnings* section for additional recommendations based on the type of specimen to scan.

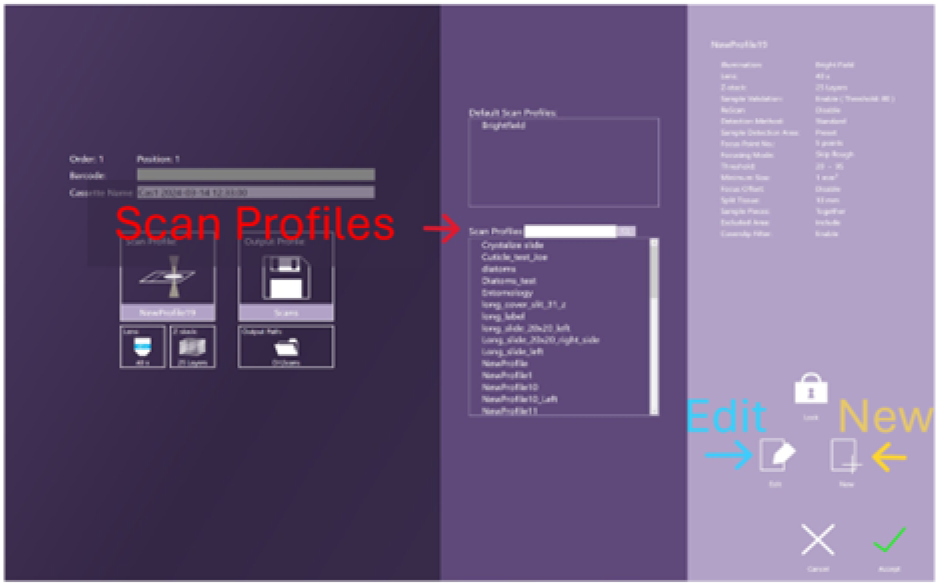

13.1 Click on “**Scan Profile Name”** to rename the profile.

13.2 Click on **Barcode:** This is to define the label area. To adjust, drag the green border to define the label area. After defining this area, **do not click "Apply"**; instead, **select "Scan Area"**.

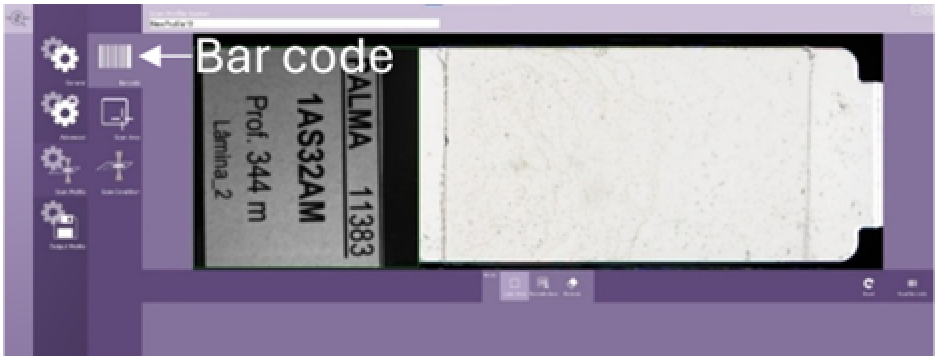

13.3 **Scan Area** defines the area to be scanned by clicking on the sample region and dragging the mouse. This area is represented in blue. The size of the selected scan area depends on the type of sample or specimen being imaged. Here you can set standard *Focus Point Numbers* and *Split tissue* for the whole cassette. Review the *Guidelines and Warnings* section for additional recommendations based on the type of specimen to scan.

Additonal notes:

- *Focus Point Numbers:* Number of automatic focal points that will be selected for focusing on the material. This feature works as quality control for appropriate specimen detection and to prevent scan failures caused by dirt or debris on the slides. The focal points are initially set automatically when defining the profile settings, varying from 1, 5, and 9 focus points. However, even when a specific number is selected, the NZ may automatically adjust the number of focus points based on material detection, and in those cases, these points can be manually modified or moved.

Before imaging, the NZ scans the entire slide and generates a focus score for each checkpoint (focus point), displayed on the monitor for quality review. The score is represented by color-coding each focus point: light green (in focus), dark green (possibly in focus), and red (out of focus). If most of the focus points are red or dark green, the scanner may not be able to proceed with image capture. In this case, focus points can be set manually to improve focus.

The number of points recommended depends on the scan area size. But we recommend setting five or nine focal points per scan area, depending on the sample. This is for better focus, especially when the scan area is divided (split tissue). Review *Guidelines* for additional recommendations based on the type of specimen to scan.

- *Split tissue:* Divides the scan area into subregions, each with its own focal plane, improving focus across uneven samples. This is particularly helpful when dealing with unevenly mounted material, as it improves the likelihood of obtaining a uniformly focused image.

This setting defines the maximum size (in mm) of the tissue piece (selected scan area) that will be focused on using the middle focal plane(see Z-stack). When the scan area is larger than the size specified in the split tissue setting (usually 5 mm), the software automatically divides it into smaller subregions and applies separate focal planes for each sub-scan area.
This setting parameter is not unique to the NZ as it is present in other slide-scanners under similar names or parameter settings.

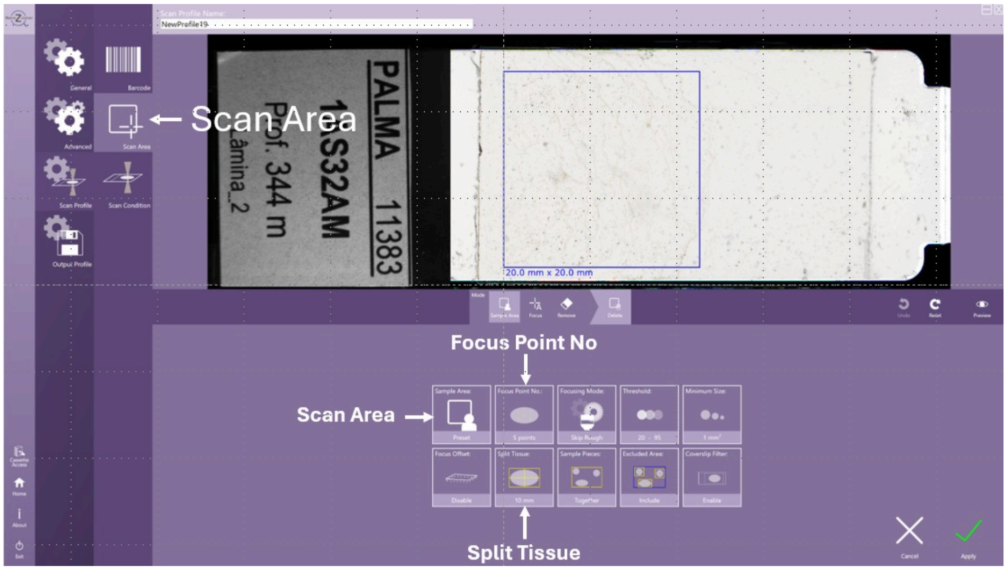

14 After selecting the “Scan Area” and defining the settings, **do not click “Apply”**; instead, **select “Scan Condition.”**

14.1 **Scan condition**: This feature captures 3D structures by imaging across multiple focal planes. Configure the magnification and Z-Stack parameters, such as Lens and Z-Stack.
Additonal notes:
- *Lens:* The NZ system uses a 20X objective with a precision optical coupler that allows scanning in two modes, 20X mode with a 0.46 µm/pixel resolution and 40X mode with 0.23 µm/pixel resolution. This feature has the advantage that the time of scanning in both modes is similar (i.e, scanning speed of a 15 × 15 µm area will take ~30s in 20X and 40X). Specimens such as insects, wood tissue, and coal ball peels can be imaged using both modes, 20X and 40X, while for microfossils, such as pollen, diatoms, and radiolarians, 40X is preferred.
- *Z-Stack:* The NZ can capture Z-stacks up to 300 µm deep, but this depth can vary among slide-scanners. Within the NZ limits, the actual scanned depth depends on both the number of layers (ranging from 1 to 301) and the spacing between the layers, which can vary from 1 to 150 µm.
Here, it is possible to select a preset condition or choose “Custom” to define a new condition. To define the number of Z-Stack planes, add an odd number in “Layers”. This is to include a central focal plane. Define the space between layers in “Distance”. The

"Offset" option moves the middle focal plane up or down if the default focus plane is not optimal.

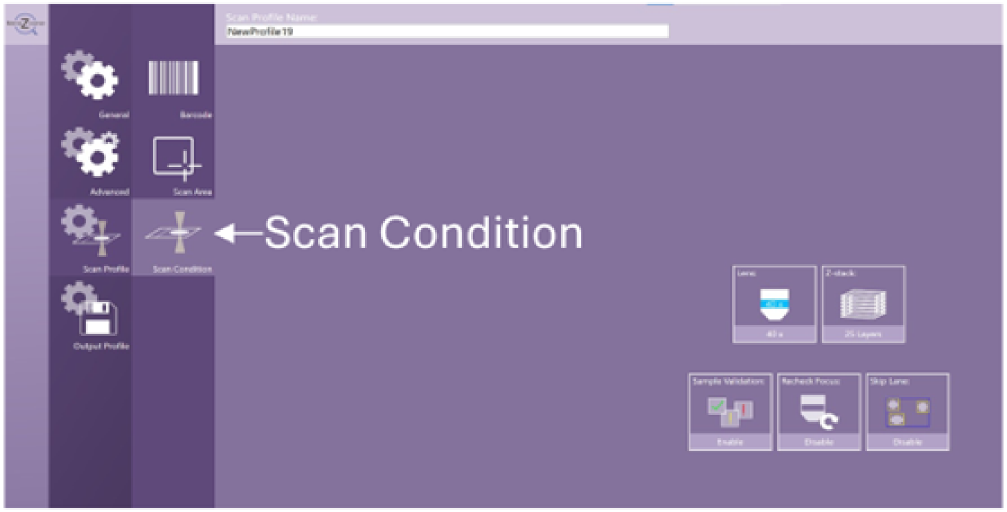

15 If the Z-stack" settings were modified when selecting "Custom", **click "Accept"** to save the changes in the profile

15.1 Click **"Apply"** to save the profile created.

Naming the samples before scanning

16 If the slides have a QR code or a barcode, naming is automatic. If there is no QR code, prepare a .txt or .csv file listing sample names in the same order as they are placed in the cassette. Do not add a title or spaces between names. The preparation of the .txt or .csv file can take between 5 to 10 minutes, depending on the number of slides that will be scanned. The maximum number of slides that can be placed in the NZ S20 cassete is 20 slides.

17 After setting the "Scan Profile", click "Import" (red square) to add the sample names from the prepared file.
If the names are not added a priori, these can be added manually in the numered area (red square) or during each slide review.

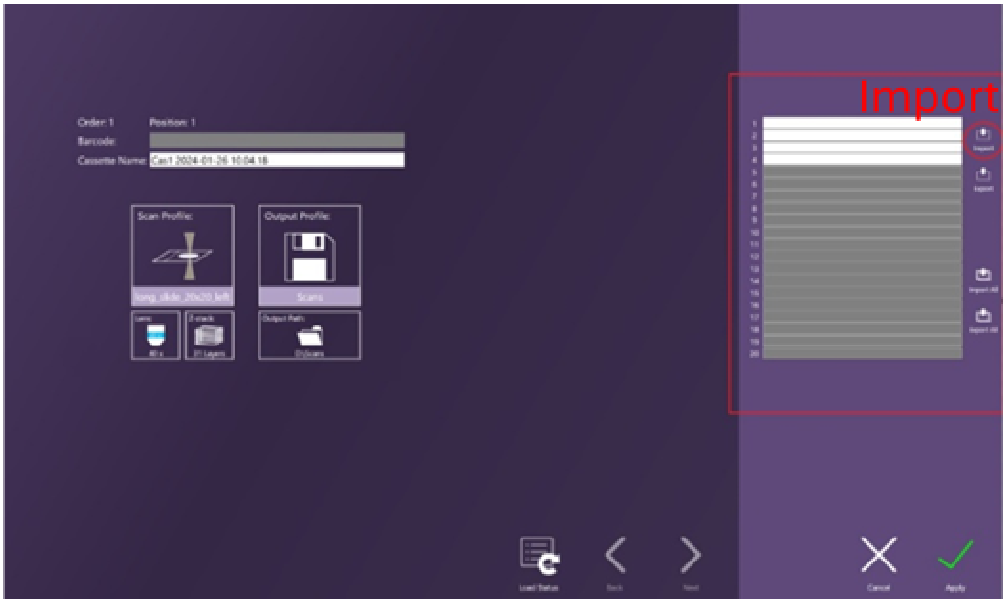

18 Click "**Apply**".

19 A message will pop up, select "**Overwrite**".

20 Select “**Slide View**” and review each slide in succession by clicking on the slide image on the left.
To modify "Sample Area" or "Focus", use the "Mode" bar (red square).
- Sample Area: Select “Sample Area” and “remove” to remove and redraw the area for scanning by clicking and dragging on the image.
- Focus: If the focal points are not optimal in position or amount, select “Focus” and “Remove”. Then, click on the Scan area selected to add new focal points. If the Scan area was modified, select “Focus” and "Auto” to set the new focal points.

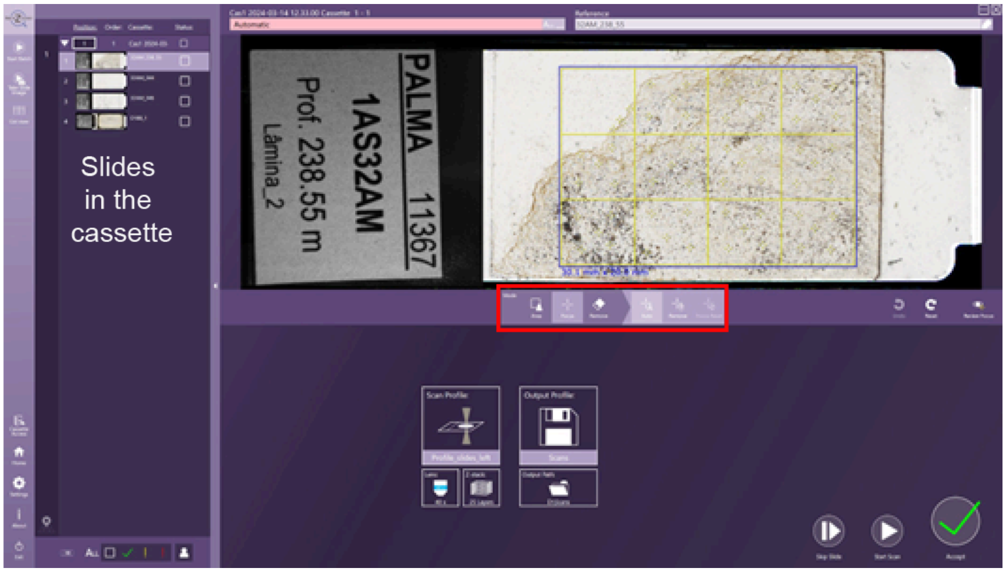

Start scanning

21 Click “**Accept**” to start the scanning.
NOTE: Before scanning begins, the NZ checks on the focal points. Light green indicates in focus, dark green is maybe in focus, and red indicates out of focus. While the first sample is scanning, other samples can be reviewed and prepared.
To review and edit the scan area and focus points, check steps 13.3.
IMPORTANT: If autofocus failed, go to "Scan Settings". In the Mode bar (step 20), select "Focus", then "Remove". Manually add focus points by clicking on the selected scan area. Finally, select "Accept".

22 During scanning, NZAcquire displays the estimated total time (Total), time already scanned (Elapsed), and time left (Remaining). Scanning progress can be monitored on the left side of the screen. Scanned samples display a yellow, green, or red symbol. Samples waiting to be scanned display an hourglass symbol.

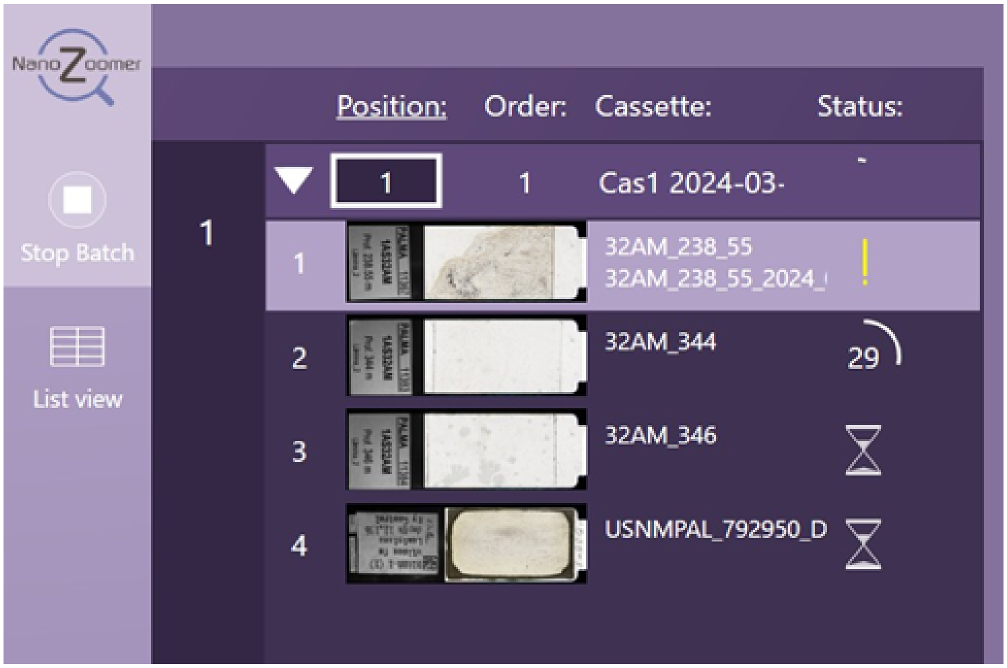

Checking images

23 Select “View slide” after each sample finishes scanning to review the final image.

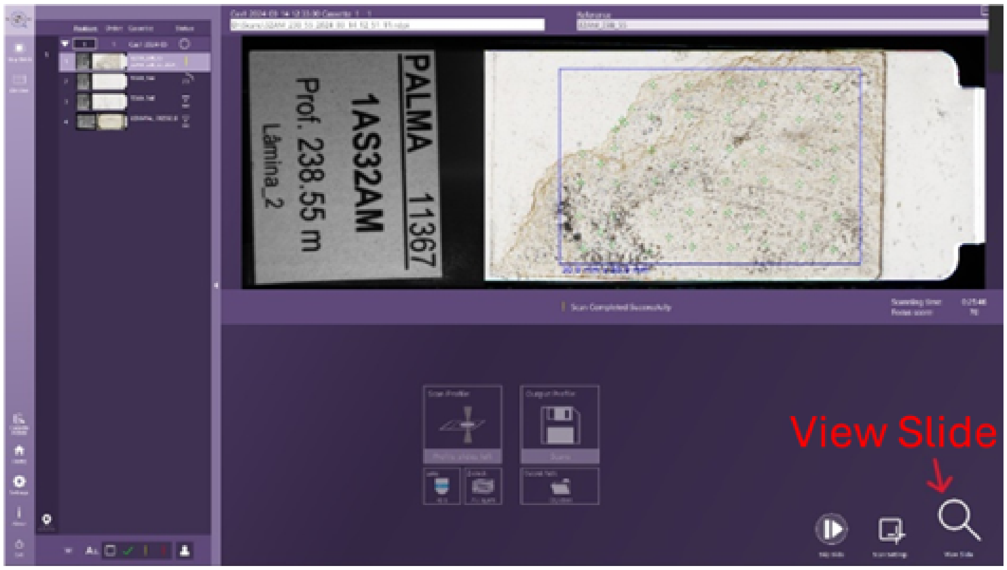

24 If image quality is acceptable, click “Accept”.

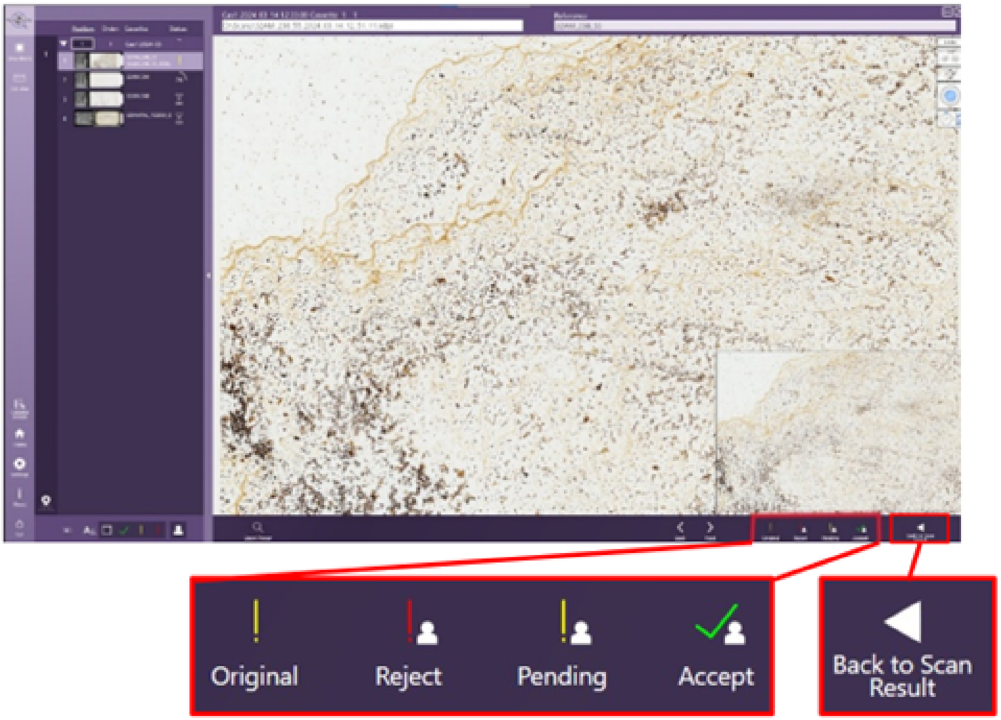

25 Select “Back to Scan Result”, then “Scan Settings” to continue scanning.

Extracting samples

26 After scanning, select “Cassette Access” to extract slides. Refer to steps 3 to 6 for cassette removal and reinsertion.

27 After reinserting the cassette, select “Scan Settings”.

28 To start a new scan, follow the protocol from **Section 2**.
To exit the software, click the “**X**” in the top right corner

Visualization and analysis of NDPI files

29 NanoZoomer Digital Pathology Images (NDPI) can be open, manage and analyze using NDP.view2, a free software created by Hamamatsu (Hamamatsu 2018) or using other free and open-access software tools created to open not only NDPI files but other propertary whole-slide images (WSI), such as: a) ImageJ Fiji, which has the NDPITools ImageJ plugin to convert the slides to standard TIFF format (Schindelin et al. 2012); and b) OpenSlide, is a C library that provides a simple interface for reading the images (Goode et al. 2013) and it can be used through different iinterfaces or platforms, such as

Python with OpenSlide-Python, through OMERO (Open Microscopy Environment), and QuPath (Open Software for Bioimage Analysis).

Accesibility of image files

30 We recommend two primary tools, Globus and OMERO to share WSI (e.g.,NDPI files), among collaborators, institutions, and collections users.
Globus: is a file transfer service that provides reliable data transfer between workstations, high-performance computer servers, data repositories, user devices, and endpoints (Globus, 2010). It excels in high-speed transfers and allows for data stream interruptions (Globus 2010). Globus allows centralized control over data and minimizes concerns about file loss risks during transfers, making it especially reliable for large-scale data sharing.
OMERO: is a free, open-source, image data management interface for viewing files on a remote server (Allan et al. 2012, 2019). OMERO allows multiple scientists to simultaneously visualize and analyze the image files, facilitating not only accessing and processing, but also sharing of large image data and metadata (Allan et al. 2012, 2019). OMERO also allows users to analyze the images without having to handle the large, original files because these are maintained by the institution housing the files (Allan et al. 2012, 2019, Jaramillo et al. 2025).

Post-processing for further analyses

31 The NDPI files (.ndpi extension) generated by the NZ, use a TIFF-like structure but differ in that each file includes a stack of multiple images (Z-stack layers), and also supports image files larger than 4 GB (OpenSlide 2010). Additionally, NDPI files are compressed in a lossy format by the scanner software, creating final files that contain all the metadata but are less than 10% of the original image size. Thus, the final size of the files from the specimens imaged here ranged from a few megabytes (MB) to several gigabytes (GB). For natural history collections, we recommend initially preserving the proprietary file formats. such as NDPIs in their original format while also saving them as an open-access New Generation File Format (NGFF), such as Hierarchical data format (HDF5) (Moore et al. 2021, The HDF Group 2021), or Zarr (Moore et al. 2021, 2023). These standardized, open formats are designed to support scalable access to large, high-resolution multi-dimensional data and associated metadata (Dworak et al. 2025, Goldberg et al. 2025). This recommendation is based on the still-evolving nature of best practices for long-term SWI preservation across institutions.

Protocol references

Schindelin J, Arganda-Carreras I, Frise E. Fiji: an open-source platform for biological-image analysis. Nat Methods. 2012;9:676–82.

Allan C, Burel J-M, Moore J, Blackburn C, Linkert M, Loynton S, et al. OMERO: flexible, model-driven data management for experimental biology. Nat Methods. 2012;9:245–53.

Allan E, Livermore L, Price B, Shchedrina O, Smith V. A Novel Automated Mass Digitisation Workflow for Natural History Microscope Slides. Biodivers Data J. 2019;7:e32342.

Dworak N, Cooper M, Fox J, Oliveira A. RAWtunda: a toll to convert a multi-channel raw image onto TIFF and OME-TIFF formats. bioRxiv. 2025.

Globus. 2010. Globus: UChicago non-profit service [Internet]. [cited 2024 May 10]. Available from: Globus.org

Goldberg I, Allan C, Burel A, Creager D, Falconi A, Hochheiser H, et al. The Open Microscopy Environment (OME) Data Model and XML file: open tools for informatics and quantitative analysis in biological imaging. Genome Biol. 2025;6:15892875

Goode A, Gilbert B, Harkes J, Jukic D, Mahadev SM. OpenSlide: A vendor-neutral software foundation for digital pathology. J Pathol Inform. 2013;4(1):2153–3539.

Hamamatsu. NDP.view2 Image viewing software [Internet]. 2018 [cited 2024 Apr 6]. Available from: https://www.hamamatsu.com/jp/en/product/life-science-and-medical-systems/digital-slide-scanner/U12388-01.html

Jaramillo C, Punyasena SW, Alba D de, Alveo R, Arcila A, Bermudez J, et al. Digitizing collections to unlock the full potential of palynology: A case study with the Smithsonian palynology collection. Plant People Planet. 2025;1–16.

Moore J, Allan C, Besson S, et al. OME-NGFF: a next-generation file format for expanding bioimaging data-access strategies. Nat Methods. 2021;18:1496–8.

Moore J, Basurto-Lozada D, Besson S, Bogovic J, J B, et al. OME-Zarr: a cloud-optimized bioimaging file format with international community support. Histochem Cell Biol. 2023;160:223–51.

OpenSlide. 2010. Hamamatsu format. OpenSlide [Internet]. 2025 [cited 2025 Sep 1]. Available from: http://openslide.org

The HDF Group. The HDF5 Library and File Format [Internet]. 2021 [cited 2026 Jan 20]. Available from: https://www.hdfgroup.org/solutions/hdf5/

Acknowledgements

We want to thank Jonathan Wingerath for facilitating the specimens of the paleobotany collection, Gene Hunt for the ostracod specimens, Torsten Dikow for the Siphonaptera specimens, Bruno de Medeiros for the Coleoptera specimens, Diana Diaz for the modern wood material, Leslie Brothers for the diatoms, Brian Huber for the radiolarians, and Ian Stocks for the specimens of Aleyrodidae.

The NDPI files of the specimens selected to demonstrate variations in scanning settings can be found in Zenodo DOIs: 10.5281/zenodo.17561743; 10.5281/zenodo.18397445; 10.5281/zenodo.18397703; and 10.5281/zenodo.18461326
